# Insights into the cardiovascular benefits of taurine: a systematic review and meta-analysis

**DOI:** 10.1186/s12937-024-00995-5

**Published:** 2024-08-15

**Authors:** Chih-Chen Tzang, Wei-Chen Lin, Long-Huei Lin, Ting-Yu Lin, Ke-Vin Chang, Wei-Ting Wu, Levent Özçakar

**Affiliations:** 1 School of Medicine, College of Medicine, National Taiwan University, Taipei, Taiwan, R.O.C.; 2 School of Physical Therapy and Graduate Institute of Rehabilitation Science, College of Medicine, Chang Gung University, Linkou, Taoyuan Taiwan, R.O.C.; 3 Department of Physical Medicine and Rehabilitation, Lo-Hsu Medical Foundation, Inc., Lotung Poh-Ai Hospital, Yilan, Taiwan; 4 Department of Physical Medicine and Rehabilitation, National Taiwan University Hospital, College of Medicine, National Taiwan University, Taipei, Taiwan; 5 Department of Physical Medicine and Rehabilitation, National Taiwan University Hospital, Bei-Hu Branch, Taipei, Taiwan; 6 Center for Regional Anesthesia and Pain Medicine, Wang-Fang Hospital, Taipei Medical University, Taipei, Taiwan; 7 Department of Physical and Rehabilitation Medicine, Hacettepe University Medical School, Ankara, Turkey

**Keywords:** Taurine, Heart failure, Cardiac function, Hypertension, Nutrition

## Abstract

**Background:**

Cardiovascular disease (CVD) remains the foremost cause of mortality globally. Taurine, an amino acid, holds promise for cardiovascular health through mechanisms such as calcium regulation, blood pressure reduction, and antioxidant and anti-inflammatory effects. Despite these potential benefits, previous studies have yielded inconsistent results. This meta-analysis of randomized controlled trials (RCTs) aims to evaluate the existing evidence on the quantitative effects of taurine on hemodynamic parameters and cardiac function grading, which are indicative of overall cardiovascular health and performance.

**Methods:**

We conducted an electronic search across multiple databases, including Embase, PubMed, Web of Science, Cochrane CENTRAL, and ClinicalTrials.gov, from their inception to January 2, 2024. Our analysis focused on key cardiovascular outcomes, such as heart rate (HR), systolic blood pressure (SBP), diastolic blood pressure (DBP), left ventricular ejection fraction (LVEF), and New York Heart Association (NYHA) Functional Classification. Meta-regression was applied to explore dose-dependent relationships based on the total taurine dose administered during the treatment period. A subgroup analysis, stratified according to the baseline disease status of patients, was also conducted.

**Results:**

The analysis included a pooled sample of 808 participants from 20 randomized controlled trials. Taurine demonstrated a significant reduction in HR (weighted mean difference [WMD] = -3.579 bpm, 95% confidence interval [CI] = -6.044 to -1.114, *p* = 0.004), SBP (WMD = -3.999 mm Hg, 95% CI = -7.293 to -0.706, *p* = 0.017), DBP (WMD: -1.435 mm Hg, 95% CI: -2.484 to -0.386, *p* = 0.007), NYHA (WMD: -0.403, 95% CI: -0.522 to -0.283, *p* < 0.001), and a significant increase in LVEF (WMD: 4.981%, 95% CI: 1.556 to 8.407, *p* = 0.004). Meta-regression indicated a dose-dependent reduction in HR (coefficient = -0.0150 per g, *p* = 0.333), SBP (coefficient = -0.0239 per g, *p* = 0.113), DBP (coefficient = -0.0089 per g, *p* = 0.110), and NYHA (coefficient = -0.0016 per g, *p* = 0.111), and a positive correlation with LVEF (coefficient = 0.0285 per g, *p* = 0.308). No significant adverse effects were observed compared to controls. In subgroup analysis, taurine significantly improved HR in heart failure patients and healthy individuals. Taurine significantly reduced SBP in healthy individuals, heart failure patients, and those with other diseases, while significantly lowered DBP in hypertensive patients It notably increased LVEF in heart failure patients and improved NYHA functional class in both heart failure patients and those with other diseases.

**Conclusions:**

Taurine showed noteworthy effects in preventing hypertension and enhancing cardiac function. Individuals prone to CVDs may find it advantageous to include taurine in their daily regimen.

**Supplementary Information:**

The online version contains supplementary material available at 10.1186/s12937-024-00995-5.

## Introduction

Cardiovascular diseases (CVDs) encompass a group of interrelated conditions, including atherosclerosis, hypertension, heart failure, cardiomyopathy, and arrhythmia. CVD is a leading cause of global mortality, accounting for approximately 17.9 million deaths in 2019, or approximately 32% of all deaths worldwide [1]. The impact of CVDs extends beyond health, imposing a significant economic burden, with the United States alone facing an estimated annual cost of $378.0 billion [2]. These conditions not only cause substantial morbidity and mortality globally, but also place a heavy financial strain on families and communities. Although primary pharmacological treatment remains the mainstay for managing CVDs, a growing emphasis is being placed on preventive measures. These include lifestyle changes such as regular exercise, maintenance of a healthy weight, and dietary supplementation [3].

Taurine, a free β-amino acid, is a highly prevalent neurotransmitter in the human nervous system, playing several crucial physiological roles. These include regulating calcium transport and homeostasis, acting as an osmolyte, and serving as a trophic factor during central nervous system development [4]. The therapeutic potential of taurine in CVDs has garnered significant interest. Research indicates that taurine influences the phosphorylation state of proteins involved in excitation–contraction coupling. It may exert inotropic effects by modulating sarcoplasmic reticular Ca^2+^ release and enhancing myofibril sensitivity to Ca^2+^ [5]. Additionally, taurine increases nitric oxide availability, which contributes to lower blood pressure by vasodilation [6]. Moreover, taurine has the potential to reduce blood pressure by inhibiting the renin–angiotensin–aldosterone system, while also showcasing antioxidative and anti-inflammatory effects [5]. Taurine exhibits anti-inflammatory properties by elevating antioxidant activity and reducing inflammatory cytokine expressions [7]. It therefore mitigates atherogenesis through several mechanisms, such as decreasing the activity of 3-hydroxy-3-methylglutaryl CoA reductase, increasing 7α-hydroxylase activity to expedite cholesterol degradation, and lowering reactive oxygen species [8].

Despite numerous clinical studies demonstrating the various health benefits of taurine, inconsistencies in outcomes present challenges in conclusively determining its effects on CVDs. This meta-analysis of randomized controlled trials (RCTs) aims to evaluate the current evidence regarding the quantitative impact of taurine on hemodynamic parameters and cardiac function grading, which are indicative of overall cardiovascular health and performance.

## Materials and methods

### General guidelines

This meta-analysis was conducted in accordance with the guidelines provided in the most recent version of the PRISMA 2020 guidelines (Table S1) [9]. The review was registered on Inplasy.com under the registration number INPLASY202410074. Independent searches were conducted by two authors (T.-C.C. and L.-W.C.) across several databases, including Embase, PubMed, Web of Science, Cochrane CENTRAL, and ClinicalTrials.gov. The search strategy employed the keywords ('taurine’ OR 'taufon') AND ('cardiovascular disease' OR 'vascular disease' OR 'hypertension' OR 'blood pressure' OR 'heart failure' OR 'atherosclerosis' OR 'arrhythmia' OR 'coronary heart disease' OR 'peripheral arterial disease'). The comprehensive search strategy is detailed in Table S2.

The search period covered the inception of each database until January 2, 2024. Supplementary Material (Table S2) provides a detailed description of the search process and a comprehensive overview of the search methodology used in this systematic review and meta-analysis. The two authors who were in charge of this search first determined the eligibility of the identified titles and abstracts by a consensus process. Other databases and reference lists of previous meta-analyses were then manually searched. After retrieving a total of 3560 studies from all sources using the provided keywords and pooling them in Endnote 21, duplicates were removed using the built-in function, reducing the number to 2428 studies. Two authors then independently screened the titles and abstracts, resulting in a kappa value of 0.81, indicating strong agreement [10]. Following a consensus discussion, 42 studies were selected for full-text assessment. The full-text screening phase yielded a kappa value of 0.84, also indicating strong agreement. Ultimately, 20 studies were deemed eligible for inclusion.

No language limitations were applied during the search, allowing the inclusion of studies published in languages other than English [10].

### Inclusion and exclusion criteria

The current meta-analysis used the following PICO (population, intervention, comparison, and outcome) settings: P, human participants; I, taurine supplementation; C, supplementation (including placebo) other than taurine; and O, parameters associated with cardiovascular function.

We applied the following inclusion criteria: (1) RCTs incorporating pure taurine and its compounds as the treatment arm, (2) inclusion of a comparative arm utilizing interventions other than taurine, and (3) trials providing available data for pre- and post-intervention assessments or evaluations of changes in one or more of the recorded outcomes.

The meta-analysis applied the following exclusion criteria: (1) non-RCTs; (2) inadequate follow-up periods that were insufficient to demonstrate results on CVDs; (3) herbal treatments without documentation of active compounds; (4) insufficient data for pre- and post-intervention endpoints; and (5) studies that lacked outcomes pertinent to the focus of interest.

### Methodological quality appraisal

We used the Cochrane risk-of-bias tool for randomized trials (RoB 2, London, United Kingdom) to assess the methodological quality of the evaluated studies, which included six main items: randomization process, intervention adherence, outcome measurement, missing outcome data, selective reporting, and overall risk of bias [11]. The RoB 2 framework offers two options for assessing intervention adherence: intention-to-treat and per-protocol evaluations. Given that most RCTs provide data only for participants who completed the entire trial course, we opted to perform a per-protocol evaluation [11].

### Outcome measurements

The main outcomes assessed in this investigation included: (1) heart rate (HR), (2) systolic blood pressure (SBP), (3) diastolic blood pressure (DBP), (4) left ventricular ejection fraction (LVEF), and (5) New York Heart Association (NYHA) Functional Classification. Additional outcomes included adverse effects. For the calculations, the number of cells with zero adverse events was adjusted to 0.5 [12].

### Data extraction and management

From the reviewed studies, two independent authors (T. C. C. and L. W. C.) extracted data, including outcome values, research design, taurine and controlled regimen details, and demographic information. To reduce the possibility of incorrect interpretation of results, the evaluators carefully examined the direction of the scale used in each trial. When data were missing from published studies, attempts were made to contact the relevant authors to acquire the original data. The process of extracting, converting, and combining results from distinct study arms using different taurine dosages was performed in accordance with the Cochrane Handbook for Systematic Reviews of Interventions and relevant medical literature [13, 14]. We extracted the outcomes reported at the conclusion of the intervention for statistical analysis if posttreatment data were available for multiple time periods.

### Statistical analyses

The present meta-analysis utilized Comprehensive Meta-Analysis software (version 3; Biostat, Englewood, NJ, United States) and employed a random-effects model [15]. This selection was based on the heterogeneity observed in the target populations across the included studies. For all numerical outcomes, the weighted mean difference (WMD) and its corresponding 95% confidence interval (CI) were computed. Odds ratios (ORs) and their associated 95% CIs were applied to analyze categorical outcomes, specifically the rates of adverse events associated with the treatment.

Examining the *I*^2^ and Cochran's Q statistics allowed us to assess the degree of heterogeneity between trials, with *I*^*2*^ values of 25%, 50%, and 75% regarded as indicating minimal, moderate, and high heterogeneity, respectively [16]. To further explore the source of heterogeneity, subgroup analyses were performed based on the baseline disease of the participants. Meta-regression was applied to assess whether there was a dose-dependent correlation between taurine and primary outcomes, specifically examining the total taurine dosage administered during the treatment period.

Sensitivity tests were performed using the one-study removal approach [12]. To evaluate the potential presence of publication bias, we examined the distribution of effect sizes on a funnel plot and assessed the statistical significance of the corresponding results using Egger's regression test [9].

### Assessment of certainty of evidence

The certainty of the evidence was assessed using the Grading of Recommendations Assessment, Development, and Evaluation (GRADE) tool [17]. This assessment classified the evidence into four categories: 'high,' 'moderate,' 'low,' and 'very low.' The classification was based on an analysis of various factors, including the risk of bias, inconsistencies, indirectness, imprecision, and potential publication bias. The evaluation was conducted independently by two reviewers, T.-C.C. and L.-W.C. In cases of discrepancies between their assessments, discussions were held, or a consensus was sought with the corresponding author (Table S4).

## Result

### Study selection

**Fig. 1:**
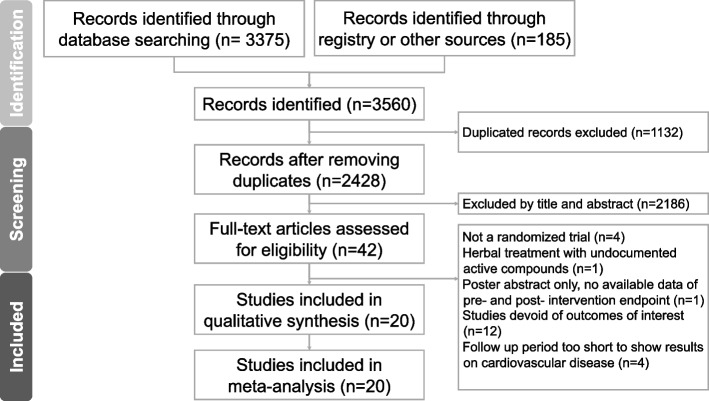
The PRISMA flow diagram of the screening and review process

The initial search yielded 3560 publications. After eliminating duplicates and conducting title/abstract screenings, 3518 articles were deemed irrelevant and were discarded. Subsequently, the full texts of the remaining 42 studies were examined. Of these, 22 articles were excluded for various reasons: four did not meet the criteria of being RCTs, one utilized herbal treatments with unverified active compounds as the intervention, one was a poster abstract lacking available data, 12 did not report outcomes aligned with our research focus, and four did not have sufficient follow-up periods to show results on CVDs (Table S3). This resulted in a total of 20 studies [18–37] being included in the final quantitative analysis (Fig. 1). Information regarding data extraction from these RCTs can be found in Tables 1 and 2.

**Table 1:** Summary of the retrieved trials investigating the effects of taurine on heart failure in the enrolled participants

First author & year	Country	Population	Participants (F/M)	Age (year)	Funding/grant/support
Azuma 1983 [33]	Japan	Congestive heart failure	58 (30/28)	38—89	N/A
Azuma 1985 [32]	Japan	Congestive heart failure	14 (5/9)	68.71 ± 9.10	Osaka University Medical School
Fujita 1987 [29]	Japan	Borderline hypertension	19 (N/A)	20—25	N/A
Azuma 1992 [34]	Japan	Idiopathic dilated cardiomyopathy	17 (6/11)	N/A	N/A
Jeejeebhoy 2002 [27]	Canada	Aortocoronary artery bypass patients	38 (2/36)	Taurine group 62 ± 11 Placebo group 69 ± 5	N/A
Spohr 2005 [20]	Denmark	Type 2 diabetes mellitus	18 (0/18)	40 ± 8	Steno Diabetes Center, Gentofte, Denmark, Aase and Ejnar Danielsens Foundation, Lyngby, Denmark
Adamchik 2010 [36]	Russia	Diastolic heart failure and metabolic syndrome	78 (59/19)	31—66	N/A
Moloney 2010 [26]	Ireland	Type 1 diabetes mellitus	19(0/19)	28.0 ± 2.0	N/A
Sedova 2010 [22]	Russia	Functional class II-III congestive heart failure	55 (N/A)	45—62	N/A
Beyranvand 2011 [31]	Iran	Heart failure with left ventricular ejection fraction less than 50%	29 (3/26)	60.57 ± 6.54	Shahid Beheshti Medical University
Roshan 2011 [23]	Iran	Heart failure	16 (N/A)	Taurine group 61.7 ± 6.4 Placebo group 60.4 ± 6.9	N/A
Gordeev 2012 [28]	Russia	Patients with functional class II-III congestive heart failure	40 (N/A)	40—70	N/A
Averin 2015 [35]	Russia	Coronary heart disease / Heart valve defects	48 (12/36)	Taurine group: 49.79 ± 1.4 Placebo group 48.65 ± 1.5	N/A
Ra 2016 [24]	Japan	Healthy men	29 (0/29)	Taurine group: 25. 4 ± 1.0 Placebo group: 25.2 ± 1.0	Japan Society for the Promotion of Science
Sun 2016 [19]	China	Prehypertensive individuals	86 (44/42)	56.75 ± 8.26	National Basic Research Program of China, National Natural Science Foundation of China
Ahmadian 2017 [37]	Iran	Heart failure	16 (N/A)	Taurine group: 60.12 ± 5.4 Placebo group: 60.13 ± 8.3	N/A
Schwarzer 2018 [21]	Austria	Patients with hepatic venous pressure gradient ≥ 12 mm Hg	22 (8/14)	52 ± 11	N/A
Esmaeili 2021 [30]	Canada	Type 2 diabetes mellitus	46 (32/14)	Taurine group: 42.74 ± 7.21 Placebo group: 43.52 ± 6.94	Tabriz University of Medical Sciences
Zaki 2021 [18]	Egypt	Peripartum cardiomyopathy	40 (40/0)	Taurine group: 31.1 ± 2.64 Placebo group: 30.85 ± 3.07	N/A
Moludi 2022 [25]	Iran	Type 2 diabetes mellitus	120 (97/23)	Taurine group: 52.13 ± 8.1 Placebo group: 53.08 ± 8.8	N/A

*N/A* Not available

**Table 2:** Summary of taurine interventions administered in the study treatment arms of the retrieved trials

First author & year	Daily taurine dose (N)	Control (N)	Population	Duration	Taurine product / manufacturer
Azuma 1983 [33]	6 g/day (58)	Matching placebo (58)	Congestive heart failure	4 weeks^a^	Not mentioned
Azuma 1985 [32]	6 g/day (14)	Matching placebo (14)	Congestive heart failure	4 weeks^a^	Not mentioned
Fujita 1987 [29]	6 g/day (10)	Matching placebo (9)	Borderline hypertension	7 days	Not mentioned
Azuma 1992 [34]	3 g/day (7)	Active placebo (10)	Idiopathic dilated cardiomyopathy	6 weeks	Taurine sachet / not mentioned
Jeejeebhoy 2002 [27]	3 g/day (20)	Matching placebo (18)	Aortocoronary artery bypass patients	35 days	MyoVive / Numico Research, Zoetermeer, The Netherlands
Spohr 2005 [20]	1.5 g/day (18)	Matching placebo (44)	Type 2 diabetes mellitus	8 weeks^a^	Taurine capsules / Not mentioned
Adamchik 2010 [36]	1 g/day (39)	Active placebo (39)	Diastolic heart failure and metabolic syndrome	12 months	Ultrasome capsules / Herbamed Ltd. (Israel)
Moloney 2010 [26]	1.5 g/day (9)	Matching placebo (10)	Type 1 diabetes mellitus	14 days^a^	Taurine tablet / Twinlab
Sedova 2010 [22]	1 g/day (32)	Active placebo (33)	Functional class II-III congestive heart failure	30 days	Taurine capsules / "dibicor" Pic-Pharma, Russia)
Beyranvand 2011 [31]	1.5 g/day (15)	Matching placebo (14)	Heart failure with left ventricular ejection fraction less than 50%	2 weeks	Taurine capsules / Solgar, Leonia, NJ, USA
Roshan 2011 [23]	1.5 g/day (7)	Matching placebo (8)	Heart failure	2 weeks	Taurine capsules / Pik Daroo Company
Gordeev 2012 [28]	1.5 g/day (20)	Active placebo (20)	Patients with functional class II-III congestive heart failure	3 months	Taurine capsules / Not mentioned
Averin 2015 [35]	0.5 g/day (24)	Matching placebo (24)	Coronary heart disease / heart valve defects	3 months	Taurine capsules / Pik-Pharma, Russian Federation
Ra 2016 [24]	6 g/day (15)	Matching placebo (14)	Healthy men	15 days	Taurine capsules / Taisho Pharmaceutical Co., Ltd., Japan
Sun 2016 [19]	1.6 g/day (42)	Matching placebo (20)	Prehypertensive individuals	12 weeks	Taurine capsules / Not mentioned
Ahmadian 2017 [37]	1.5 g/day (8)	Matching placebo (8)	Heart failure	2 weeks	Taurine capsules / Solgar, Leonia, NJ, USA
Schwarzer 2018 [21]	6 g/day (12)	Matching placebo (10)	Patients with hepatic venous pressure gradient ≥ 12 mm Hg	4 weeks	Taurine capsules / Not mentioned
Esmaeili 2021 [30]	3 g/day (23)	Matching placebo (23)	Type 2 diabetes mellitus	8 weeks	Taurine capsules / Karen Pharmaceutical Co
Zaki 2021 [18]	0.6 g/day (20)	Comparable placebo (18)	Peripartum cardiomyopathy	5 days	10 ml/kg taurine solution 10% (Aminoven®, Fresenius‑Kabi, Egypt)
Moludi 2022 [25]	3 g/day (60)	Matching placebo (60)	Type 2 diabetes mellitus	8 weeks	Taurine capsules / Karen Food Supplement Co., Iran

^a^treatment period of placebo or taurine in a cross-over study

### Study characteristic

The characteristics of the 20 included RCTs are summarized in Table 1. The included studies were conducted between 1985–2021, in Russia, Iran, Japan, Canada, Ireland, Austria, Denmark, China, and Egypt. A total of 808 participants were assigned to the taurine and control groups within the eligible studies. The participants’ ages ranged from 20 to 89 years, and the baseline health status differed between the studies, including healthy participants, heart failure, coronary heart disease, heart valve defects, idiopathic dilated cardiomyopathy, aortocoronary artery bypass, metabolic syndrome, hypertension, and prehypertensive individuals.

### Quality assessment

Fourteen studies [19, 22–24, 27–29, 31–37] did not provide allocation concealment details, and one study [28] did not mention whether the participants were aware of the intervention; thus, they were at some risk of bias. The other six studies [18, 20, 21, 25, 26, 30] had a low risk of bias, and none of the studies had a high risk of bias (Fig. S1, Table 3).

**Table 3:** Detailed quality assessment of included studies using Cochrane risk of bias 2 tool

First Author	Year	Randomization process	Intervention adherence	Missing outcome data	Outcome measurement	Selective reporting	Overall RoB
Azuma	1983	S^a^	L	L	L	L	S
Azuma	1985	S^a^	L	L	L	L	S
Fujita	1987	S^a^	L	L	L	L	S
Azuma	1992	S^a^	L	L	L	L	S
JeeJeebhoy	2002	S^a^	L	L	L	L	S
Spohr	2005	L	L	L	L	L	L
Adamchik	2010	S^a^	L	L	L	L	S
Moloney	2010	L	L	L	L	L	L
Sedova	2010	S^a^	L	L	L	L	S
Beyranvand	2011	S^a^	L	L	L	L	S
Roshan	2011	S^a^	L	L	L	L	S
Gordeev	2012	S^a^	S^b^	L	L	L	S
Averin	2015	S^a^	L	L	L	L	S
Ra	2016	S^a^	L	L	L	L	S
Sun	2016	S^a^	L	L	L	L	S
Ahmadian	2017	S^a^	L	L	L	L	S
Schwarzer	2018	L	L	L	L	L	L
Esmaeili	2021	L	L	L	L	L	L
Zaki	2021	L	L	L	L	L	L
Moludi	2022	L	L	L	L	L	L

*H* High risk of bias, *L* Low risk of bias, *RoB* Risk of bias, *S* Some risk of bias

^a^The studies did not provide allocation concealment details

^b^The study did not mention whether participants are aware of the intervention

### Main Outcome

#### Effects of Taurine on HR

**Fig. 2:**
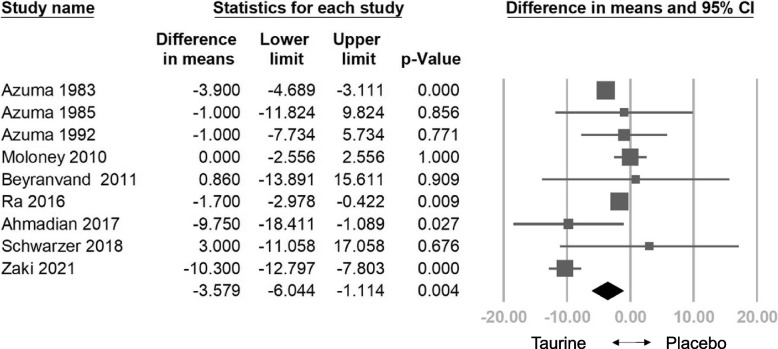
Forest plot of overall effects of taurine on heart rate (HR)

The combined effect size indicated a significant decrease in HR with taurine compared to the control group (WMD: -3.579 bpm, 95% CI: -6.044 to -1.114, *p* = 0.004, *I*^2^ = 83.394) (Fig. 2). Sensitivity analysis employing the one-study removal method consistently demonstrated the significant effect of taurine on HR reduction (Fig. S2). Meta-regression analysis indicated a correlation between taurine administration and decreased HR (coefficient = -0.0150 per g, 95% CI: -0.0458 to 0.0155, *p* = 0.333) (Fig. S3). Subgroup analysis on HR indicated that taurine had the most significant effect on treating heart failure patients (WMD: -3.898 bpm, 95% CI = -4.679 to -3.116, *p* = 0.000). It also showed a significant effect on the healthy subgroup (WMD: -1.700 bpm, 95% CI = -2.978 to -0.422, *p* = 0.009). However, it showed an insignificant effect on the other disease subgroup (WMD: -6.197 bpm, 95% CI = -15.248 to 2.853, *p* = 0.180) and diabetes subgroup (WMD: 0.000 bpm, 95% CI = -2.556 to 2.556, *p* = 1.000) (Fig.S4).

#### Effects of Taurine on SBP/DBP

**Fig. 3:**
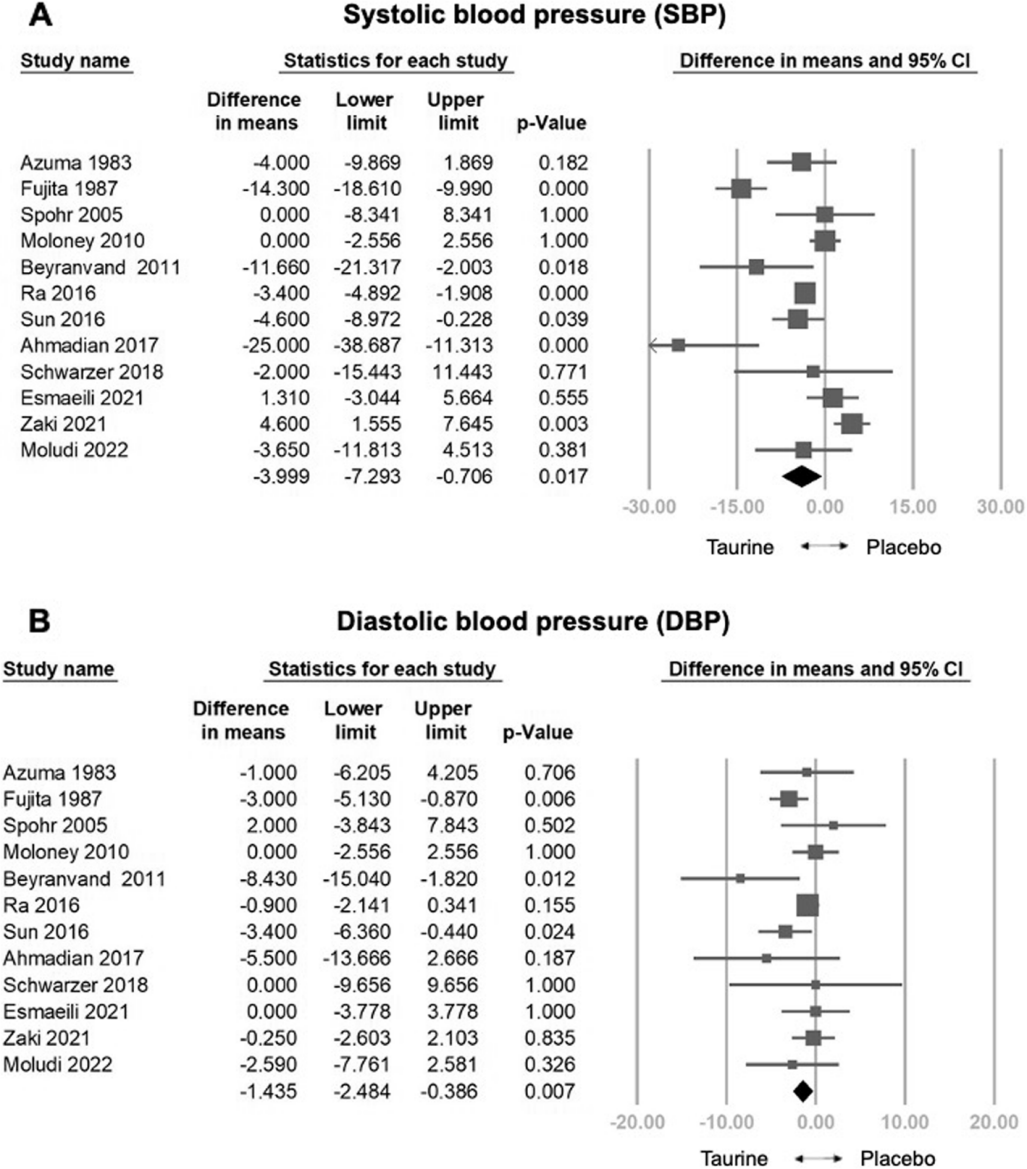
Forest plot of overall effects of taurine on systolic blood pressure (SBP) and diastolic blood pressure (DBP)

The combined effect size indicated a significant decrease in SBP with taurine compared to the control group (WMD: -3.999 mm Hg, 95% CI: -7.293 to -0.706, *p* = 0.017, *I*^2^ = 84.949) (Fig. 3a). Sensitivity analysis employing the one-study removal method consistently demonstrated a significant effect of taurine on SBP reduction (Fig. S5a). Furthermore, meta-regression analysis indicated a correlation between taurine administration and decreased SBP (coefficient = -0.0239 per g, 95% CI: -0.0535 to 0.0057, *p* = 0.113) (Fig. S6a). Subgroup analysis on SBP indicated that taurine had the most significant positive effect on treating the healthy subgroup (WMD: -3.400 mm Hg, 95% CI = -4.892 to -1.908, *p* = 0.000), an opposite effect on the other disease subgroup (WMD: 4.600 mm Hg, 95% CI = 1.555 to 7.645, *p* = 0.003), and a positive effect on heart failure patients (WMD: -9.817 mm Hg, 95% CI = -18.575 to -1.060, *p* = 0.028). However, it showed an insignificant effect on the hypertension (WMD: -9.457 mm Hg, 95% CI = -18.963 to 0.049, *p* = 0.051) and diabetes subgroup (WMD: 0.061 mm Hg, 95% CI = -2.001 to 2.123, *p* = 0.954) (Fig.S7).

The combined effect size indicated a significant decrease in DBP with taurine compared to the control group (WMD: -1.435 mm Hg, 95% CI: -2.484 to -0.386, *p* = 0.007, *I*^2^ = 21.556) (Fig. 3b). Sensitivity analysis employing the one-study removal method consistently demonstrated the significant effect of taurine on DBP reduction (Fig. S5b). Meta-regression analysis indicated a correlation between taurine administration and decreased DBP (coefficient = -0.0089 per g, 95% CI: -0.0198 to 0.0020, *p* = 0.110) (Fig. S6b). Subgroup analysis on DBP indicated that taurine had the most significant effect on treating hypertension (WMD: -3.137 mm Hg, 95% CI = -4.865 to -1.408, *p* = 0.000). It also showed some effects on heart failure patients (WMD: -3.758 mm Hg, 95% CI = -7.680 to 0.165, *p* = 0.060). However, it showed an insignificant effect on the healthy subgroup (WMD: -0.900 mm Hg, 95% CI = -2.141 to 0.341, *p* = 0.155), the other disease subgroup (WMD: -0.250 mm Hg, 95% CI = -2.603 to 2.103, *p* = 0.835) and the diabetes subgroup (WMD: -0.132 mm Hg, 95% CI = -1.990 to 1.726, *p* = 0.889) (Fig.S8).

#### Effects of Taurine on LVEF

**Fig. 4:**
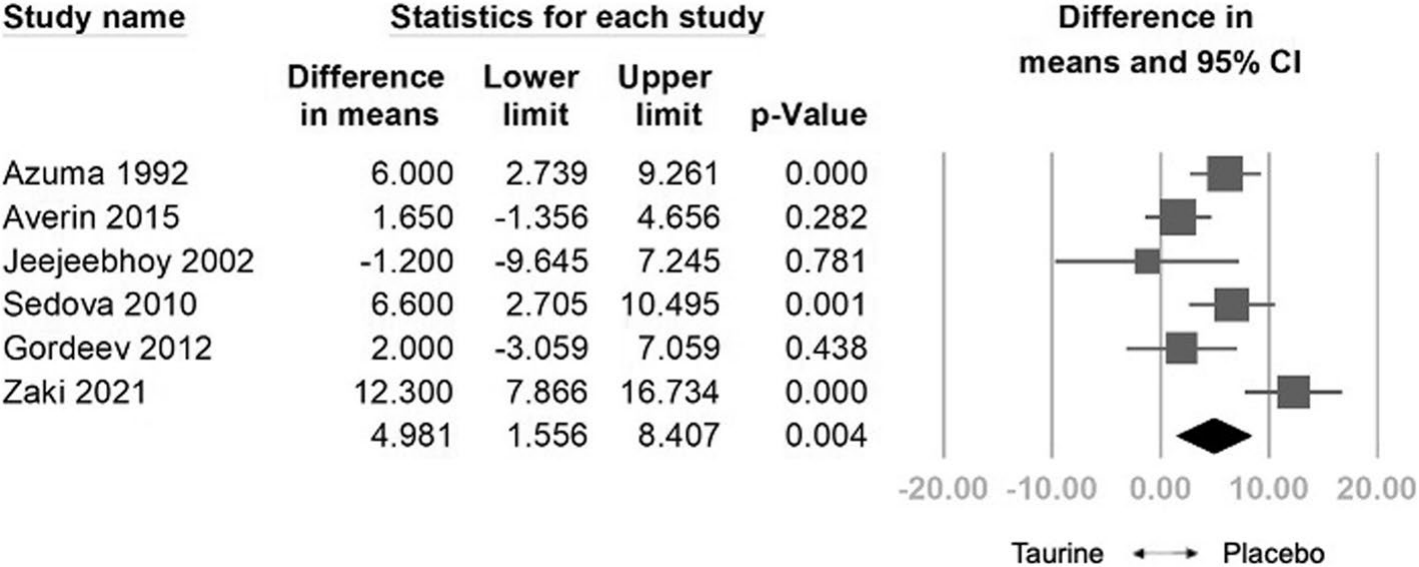
Forest plot of overall effects of taurine on left ventricular ejection fraction (LVEF)

The combined effect size indicated a significant increase in LVEF in taurine compared to the control group (WMD: 4.981%, 95% CI: 1.556 to 8.407, *p* = 0.004, *I*^2^ = 74.509) (Fig. 4). Sensitivity analysis employing the one-study removal method consistently demonstrated a significant effect of taurine on LVEF (Fig. S9). Meta-regression analysis indicated a positive correlation between taurine administration and increased LVEF (coefficient = 0.0285 per gram, 95% CI: -0.0263 to 0.0832, *p* = 0.308) (Fig. S10). Subgroup analysis on LVEF indicated that taurine had the most significant effect on treating heart failure patients (WMD: 5.370%, 95% CI = 2.982 to 7.757, *p* = 0.000). However, it showed an insignificant effect on the other disease subgroup (WMD: 4.609%, 95% CI = -3.510 to 12.728, *p* = 0.266) (Fig.S11).

#### Effects of Taurine on NYHA

**Fig. 5:**
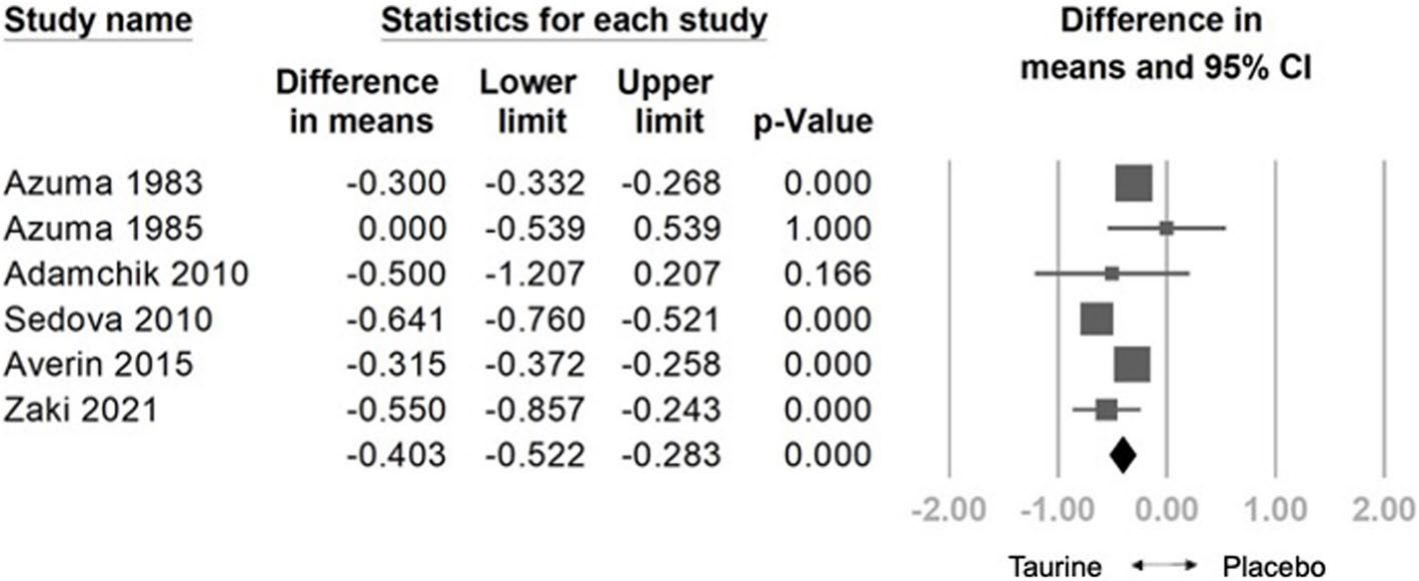
Forest plot of overall effects of taurine on New York Heart Association Functional Classification (NYHA)

The combined effect size indicated a significant decrease in NYHA with taurine compared to the control group (WMD: -0.403, 95% CI: -0.522 to -0.283, *p* < 0.001, *I*^2^ = 84.785) (Fig. 5). Sensitivity analysis employing the one-study removal method consistently demonstrated the significant effect of taurine on NYHA reduction (Fig S12). Meta-regression analysis indicated a negative relationship between taurine administration and decreased NYHA (coefficient = -0.0016 per gram, 95% CI: -0.0035 to 0.0004,* p* = 0.111) (Fig. S13). Subgroup analysis on NYHA indicated that taurine had the most significant effect on treating the other disease subgroup (WMD: -0.356, 95% CI = -0.484 to -0.227, *p* = 0.000). It also showed significant effect on heart failure patients (WMD: -0.383, 95% CI = -0.680 to -0.085, *p* = 0.012). (Fig.S14).

### Publication bias

Funnel plot analysis of all the investigated outcomes (HR, SBP, DBP, LVEF, and NYHA classification) indicated no evidence of publication bias. The distribution effect sizes were symmetric, as confirmed by Egger's regression test, with *p* values exceeding 0.5 for all outcomes (*p* = 0.934, *p* = 0.439, *p* = 0.423, *p* = 0.940, and *p* = 0.383, respectively) (Fig. S14-S18).

### Additional outcomes

#### Adverse effects

This meta-analysis examining the rates of treatment-associated adverse effects indicated no statistically significant differences between the taurine and control groups (OR = 1.328, 95% CI = 0.663 to 2.663, *p* = 0.424) (Fig.S19).

## Discussion

The principal result of this meta-analysis was that taurine supplementation leads to a significant reduction in HR, SBP, DBP, and NYHA classification, along with an improvement in LVEF. Meta-regression analysis demonstrated a noteworthy dose-dependent relationship between decreased HR and DBP.

Compared to the control group, taurine significantly reduced HR. As demonstrated in the Framingham study, a reduction in HR was linked to a decreased risk of CVDs and lower mortality rates, particularly in individuals with compromised cardiac function [38]. This decrease in HR was significantly related to improvements in LVEF and changes in the structure of the left ventricle [39]. In addition, RCTs have shown that short-term taurine supplementation can effectively reduce HR [40]. In the subgroup of patients with heart failure, taurine has been shown to significantly benefit cardiac function, which is likely caused by the elimination of compensatory tachycardia secondary to reduced ejection fraction.

Moreover, taurine significantly reduced SBP and DBP. The antihypertensive effects of taurine involve multiple mechanisms, including the improvement of endothelium-dependent vasodilation by restoring redox balance, increasing hydrogen sulfide levels [19], and enhancing nitric oxide availability [41]. Administration of taurine has also been shown to upregulate the expression of H2S-synthesizing enzymes and decrease vascular TRPC3 expression. This indicates that taurine improves vascular tone by targeting the H2S-mediated inhibition of TRPC3-induced calcium influx [19]. These findings align with a previous meta-analysis by Waldron et al. [42], which included seven trials and reported a decrease in both SBP (Hedges’ g = − 0.70, 95% CI: − 0.98 to − 0.41, *p* < 0.0001) and DBP (Hedges’ g = − 0.62, 95% CI: − 0.91 to − 0.34, *p* < 0.0001). Subgroup analysis of the SBP data revealed that taurine exhibited its most significant effects in healthy individuals, various disease subgroups, and patients with heart failure. This can be attributed to taurine’s vasodilatory properties, which tend to be effective across a broad range of individuals. However, its impact is diminished in patients with hypertension and diabetes, who generally have elevated baseline SBP [43]. Conversely, the DBP data demonstrated that taurine effectively lowers DBP in patients with hypertension and heart failure. DBP, a crucial measure of vascular health, indicates the pressure in the arteries during the heart's resting phase between beats. Taurine enhances vascular function, which in turn lowers DBP [44]. This is particularly beneficial for managing conditions such as hypertension and heart failure, where diastolic dysfunction poses a significant challenge.

Taurine significantly enhanced LVEF and reduced NYHA grading. In a previous meta-analysis conducted by McGurk et al. [45] that encompassed three studies, there was a tendency towards LVEF improvement, although it did not reach statistical significance (standardized mean difference = 0.25, 95% CI -0.38 to 0.89). Taurine exerts positive inotropic effects on the heart by stimulating the calcium-activated ATPase pump, aiding in calcium regulation within muscle cells, and counteracting disruptions in Ca^2+^ homeostasis commonly observed in heart failure [5]. Additionally, taurine enhances cardiac function through the stimulation of adenylate cyclase and phosphodiesterase, potentially increasing cyclic adenosine monophosphate turnover in the heart [46]. The antioxidant properties of taurine are crucial in mitigating oxidative stress, which is often elevated in individuals with compromised cardiovascular function [47]. Studies have also shown that taurine prevents ischemia-induced apoptosis in cardiomyocytes by inactivating caspase-9 and caspase-3, and inhibiting the formation of the Apaf-1/caspase-9 apoptosome, ultimately protecting the cardiomyocytes [48]. In studies involving patients with heart failure, most participants were classified as NYHA class 2 or 3 and displayed either moderate (40–50%) or decreased (< 40%) LVEF. These results highlight the potential of taurine in improving cardiac function, particularly in patients with moderately severe heart failure. Subgroup analysis of LVEF and NYHA classification data indicates that taurine is effective in patients with heart failure due to their potential for significant improvement and taurine's positive inotropic effect. Additionally, the "other diseases" subgroup, which includes conditions like cardiomyopathy and coronary artery disease—often associated with some level of heart failure—suggests that these patients might also benefit from taurine intervention.

The United States Food and Drug Administration categorizes taurine as a substance "generally recognized as safe" [49]. There were no significant negative effects of taurine in our study, despite the varying range of doses (1.5—6 g/day) and lengths of supplementation periods (5–90 days). All negative effects of taurine (potentially) were moderate and transient.

Although some endpoints included in this study have been examined previously [42, 45], our meta-analysis is the first to compile sufficient data to suggest a dose–response relationship. However, several factors may explain the lack of statistically significant dose-dependent effects of taurine. Firstly, the relatively limited cellular transport capacity for taurine may result in reaching a saturation point, thus diminishing its efficacy at higher doses. [50]. It is also important to note that the kidneys exhibit both high clearance and excretion capabilities, as the clearance rate of orally administered taurine has been reported to be dose-dependent [51].

This study has several limitations. Firstly, a random-effects model, which assumes heterogeneity across studies, was employed to account for differences in patient baseline characteristics, variable doses, and trial durations, as opposed to a fixed-effect model. However, this approach may introduce biased estimates due to substantial heterogeneity, exacerbate publication bias, and disproportionately influence smaller studies with variable results. To address these issues, we conducted subgroup analyses, and dose dependent meta-regression to investigate the source of heterogeneity. Additionally, 14 out of 20 studies did not provide details on allocation concealment, which, according to the Cochrane risk-of-bias tool, raises concerns about potential bias. Lastly, the short follow-up duration in most included studies precludes the assessment of the long-term effects of taurine on heart failure and hypertension, ultimately limiting our ability to provide a comprehensive view of its potential benefits.

Future research should investigate combination therapies of taurine with other interventions, such as examining the effects of Camelina sativa oil in conjunction with taurine on atherogenesis [52], and provide practical recommendations regarding taurine supplementation, including optimal dosage and duration. Given the current lack of practical guidelines for taurine supplementation, we offer explicit recommendations based on existing evidence. Considering taurine's safety profile and its beneficial effects on CVDs and metabolic disorders [6], we suggest a dosage of up to 6 g per day for several months as potentially beneficial for patients with underlying cardiovascular conditions and metabolic disorders. However, the applicability to broader populations is uncertain due to the varied health statuses of participants in the included studies, and practitioners should be mindful of the observed lack of a significant dose-dependent response and adopt personalized treatment approaches tailored to individual patient needs.

## Conclusion

Taurine supplementation significantly reduces HR, SBP, DBP, and NYHA classification, while improving LVEF, especially in patients with heart failure. It is safe and effective for cardiovascular health.

### Supplementary Information

Supplementary Material 1

## Data Availability

No datasets were generated or analysed during the current study.

## Data availability

No datasets were generated or analysed during the current study.
