# Supplementary material for: Insights into the cardiovascular benefits of taurine: a systematic review and meta-analysis
Source: Nutr J. 2024 Aug 15;23:93. doi: 10.1186/s12937-024-00995-5 (PMC11325608; doi:10.1186/s12937-024-00995-5)
Supplement: Supplementary file 1 — Supplementary Material 1 [file 12937_2024_995_MOESM1_ESM.docx]

**Supplemental Material**

**Table S1**. PRISMA Checklist

| **Section and Topic** | **#** | **Checklist item** | **Location** |
| --- | --- | --- | --- |
| **TITLE** | | |  |
| Title | 1 | Identify the report as a systematic review. | Title |
| **ABSTRACT** | | |  |
| Abstract | 2 | See the PRISMA 2020 for Abstracts checklist. | Abstract |
| **INTRODUCTION** | | |  |
| Rationale | 3 | Describe the rationale for the review in the context of existing knowledge. | Introduction |
| Objectives | 4 | Provide an explicit statement of the objective(s) or question(s) the review addresses. | Introduction |
| **METHODS** | | |  |
| Eligibility criteria | 5 | Specify the inclusion and exclusion criteria for the review and how studies were grouped for the syntheses. | Methods |
| Information sources | 6 | Specify all databases, registers, websites, organisations, reference lists and other sources searched or consulted to identify studies. Specify the date when each source was last searched or consulted. | Methods |
| Search strategy | 7 | Present the full search strategies for all databases, registers and websites, including any filters and limits used. | Methods, Table S2 |
| Selection process | 8 | Specify the methods used to decide whether a study met the inclusion criteria of the review, including how many reviewers screened each record and each report retrieved, whether they worked independently, and if applicable, details of automation tools used in the process. | Methods |
| Data collection process | 9 | Specify the methods used to collect data from reports, including how many reviewers collected data from each report, whether they worked independently, any processes for obtaining or confirming data from study investigators, and if applicable, details of automation tools used in the process. | Methods |
| Data items | 10a | List and define all outcomes for which data were sought. Specify whether all results that were compatible with each outcome domain in each study were sought (e.g., for all measures, time points, analyses), and if not, the methods used to decide which results to collect. | Methods |
|  | 10b | List and define all other variables for which data were sought (e.g., participant and intervention characteristics, funding sources). Describe any assumptions made about any missing or unclear information. | Methods, Table 1-2 |
| Study risk of bias assessment | 11 | Specify the methods used to assess risk of bias in the included studies, including details of the tool(s) used, how many reviewers assessed each study and whether they worked independently, and if applicable, details of automation tools used in the process. | Methods |
| Effect measures | 12 | Specify for each outcome the effect measure(s) (e.g., risk ratio, mean difference) used in the synthesis or presentation of results. | Methods |
| Synthesis methods | 13a | Describe the processes used to decide which studies were eligible for each synthesis (e.g., tabulating the study intervention characteristics and comparing against the planned groups for each synthesis (item #5)). | Methods, Figure 1,  Table 1-2, Table S3 |
|  | 13b | Describe any methods required to prepare the data for presentation or synthesis, such as handling of missing summary statistics, or data conversions. | Methods |
|  | 13c | Describe any methods used to tabulate or visually display results of individual studies and syntheses. | Methods |
|  | 13d | Describe any methods used to synthesize results and provide a rationale for the choice(s). If meta-analysis was performed, describe the model(s), method(s) to identify the presence and extent of statistical heterogeneity, and software package(s) used. | Methods |
|  | 13e | Describe any methods used to explore possible causes of heterogeneity among study results (e.g., subgroup analysis, meta-regression). | Methods |
|  | 13f | Describe any sensitivity analyses conducted to assess robustness of the synthesized results. | Methods |
| Reporting bias assessment | 14 | Describe any methods used to assess risk of bias due to missing results in a synthesis (arising from reporting biases). | Methods, Figure S1,  Table 3 |
| Certainty assessment | 15 | Describe any methods used to assess certainty (or confidence) in the body of evidence for an outcome. | Methods |
| **RESULTS** | | |  |
| Study selection | 16a | Describe the results of the search and selection process, from the number of records identified in the search to the number of studies included in the review, ideally using a flow diagram. | Results, Figure 1,  Table S2-S3 |
|  | 16b | Cite studies that might appear to meet the inclusion criteria, but which were excluded, and explain why they were excluded. | Results, Table S3 |
| Study characteristics | 17 | Cite each included study and present its characteristics. | Results, Table 1-2 |
| Risk of bias | 18 | Present assessments of risk of bias for each included study. | Table 3, Figure S1 |
| Results of individual studies | 19 | For all outcomes, present, for each study: (a) summary statistics for each group (where appropriate) and (b) an effect estimates and its precision (e.g., confidence/credible interval), ideally using structured tables or plots. | Figure 2-5, Figure S2-S9, S14 |
| Results of syntheses | 20a | For each synthesis, briefly summarise the characteristics and risk of bias among contributing studies. | Results, Table 3 |
|  | 20b | Present results of all statistical syntheses conducted. If meta-analysis was done, present for each the summary estimate and its precision (e.g., confidence/credible interval) and measures of statistical heterogeneity. If comparing groups, describe the direction of the effect. | Results, Figure 2-5, Figure S2-S9, S14 |
|  | 20c | Present results of all investigations of possible causes of heterogeneity among study results. | Results, Figure 2-5, Figure S2-S9, S14 |
|  | 20d | Present results of all sensitivity analyses conducted to assess the robustness of the synthesized results. | Results, Figure S2, S4, S6, S8 |
| Reporting biases | 21 | Present assessments of risk of bias due to missing results (arising from reporting biases) for each synthesis assessed. | Table 3, Figure S1 |
| Certainty of evidence | 22 | Present assessments of certainty (or confidence) in the body of evidence for each outcome assessed. | Figure 2-5, Figure S2-S9, S14 |
| **DISCUSSION** | | |  |
| Discussion | 23a | Provide a general interpretation of the results in the context of other evidence. | Discussion |
|  | 23b | Discuss any limitations of the evidence included in the review. | Discussion |
|  | 23c | Discuss any limitations of the review processes used. | Discussion |
|  | 23d | Discuss implications of the results for practice, policy, and future research. | Discussion |
| **OTHER INFORMATION** | | |  |
| Registration and protocol | 24a | Provide registration information for the review, including register name and registration number, or state that the review was not registered. | Methods |
|  | 24b | Indicate where the review protocol can be accessed, or state that a protocol was not prepared. | Methods, Table S2-S3 |
|  | 24c | Describe and explain any amendments to information provided at registration or in the protocol. | Methods, Table S2-S3 |
| Support | 25 | Describe sources of financial or non-financial support for the review, and the role of the funders or sponsors in the review. | Funding |
| Competing interests | 26 | Declare any competing interests of review authors. | Conflicts of Interest |
| Availability of data, code and other materials | 27 | Report which of the following are publicly available and where they can be found: template data collection forms; data extracted from included studies; data used for all analyses; analytic code; any other materials used in the review. | Results, Table S2-S3 |

**Table S2.** Keywords and search results in different databases

| Database | Keyword | Filter | Date | Results |
| --- | --- | --- | --- | --- |
| PubMed | ('taurine' OR 'taufon') AND ('cardiovascular disease' OR 'vascular disease' OR 'hypertension' OR 'blood pressure' OR 'heart failure' OR 'atherosclerosis' OR 'arrhythmia' OR 'coronary heart disease' OR 'peripheral arterial disease' OR 'stroke ') | NA | January 2nd,  2024 | 2241 |
| Embase | ('taurine' OR 'taufon') AND ('cardiovascular disease' OR 'vascular disease' OR 'hypertension' OR 'blood pressure' OR 'heart failure' OR 'atherosclerosis' OR 'arrhythmia' OR 'coronary heart disease' OR 'peripheral arterial disease' OR 'stroke ') | Randomized controlled trial | January 2nd,  2024 | 70 |
| Cochrane CENTRAL | ('taurine' OR 'taufon') AND ('cardiovascular disease' OR 'vascular disease' OR 'hypertension' OR 'blood pressure' OR 'heart failure' OR 'atherosclerosis' OR 'arrhythmia' OR 'coronary heart disease' OR 'peripheral arterial disease' OR 'stroke') | Trials | January 2nd,  2024 | 115 |
| Web of Science | ('taurine' OR 'taufon') AND ('cardiovascular disease' OR 'vascular disease' OR 'hypertension' OR 'blood pressure' OR 'heart failure' OR 'atherosclerosis' OR 'arrhythmia' OR 'coronary heart disease' OR 'peripheral arterial disease' OR 'stroke') | NA | January 2nd,  2024 | 1606 |
| ClinicalTrials.gov | ('taurine' OR 'taufon') AND ('cardiovascular disease' OR 'vascular disease' OR 'hypertension' OR 'blood pressure' OR 'heart failure' OR 'atherosclerosis' OR 'arrhythmia' OR 'coronary heart disease' OR 'peripheral arterial disease' OR 'stroke') | Condition  or disease | January 2nd,  2024 | 11 |

NA: not applicable

**Table S3.** Excluded studies and reasons

| **Citations** | **Reasons** |
| --- | --- |
| Ahn, C. S. (2009). Effect of taurine supplementation on plasma homocysteine levels of the middle-aged Korean women. *Adv Exp Med Biol*, 643, 415-422. | Not a randomized trial |
| Fennessy, F. M., Moneley, D. S., Wang, J. H., Kelly, C. J., & Bouchier-Hayes, D. J. (2003). Taurine and vitamin C modify monocyte and endothelial dysfunction in young smokers. *Circulation*, 107(3), 410-415. | Not a randomized trial |
| Montanini, R., Zibetti, A., & Gasco, P. (1971). [Preliminary observations on the use of taurine in the treatment of cerebrovascular diseases]. *Clin Ter*, 59(4), 321-329. (Osservazioni preliminari sull'impiego della taurina nel trattamento delle vasculopatie cerebrali.) | Not a randomized trial |
| Montanini, R., & Gasco, P. (1974). [Taurine in the treatment of diffuse cerebral arteriopathies. Clinical and electroencephalographic observations and psychological tests]. *Clin Ter*, 71(5), 427-436. | Not a randomized trial |
| Basrai, M., Schweinlin, A., Menzel, J., Mielke, H., Weikert, C., Dusemund, B., Putze, K., Watzl, B., Lampen, A., & Bischoff, S. C. (2019). Energy drinks induce acute cardiovascular and metabolic changes pointing to potential risks for young adults: A randomized controlled trial [Article]. *Journal of Nutrition*, 149(3), 441-450. | Follow up period too short to show results on cardiovascular disease |
| Speck, N. E., Michalak, M., Dreier, K., Babst, D., Lardi, A. M., & Farhadi, J. (2023). Effect of the Red Bull Energy Drink on Perfusion-Related Variables in Women Undergoing Microsurgical Breast Reconstruction: Protocol and Analysis Plan for a Prospective, Multicenter Randomized Controlled Trial. *JMIR Res Protoc,* 12, e38487. | Follow up period too short to show results on cardiovascular disease |
| Svatikova, A., Covassin, N., Somers, K. R., Somers, K. V., Soucek, F., Kara, T., & Bukartyk, J. (2015). A Randomized Trial of Cardiovascular Responses to Energy Drink Consumption in Healthy Adults. JAMA, 314(19), 2079-2082. | Follow up period too short to show results on cardiovascular disease |
| Olszanecka, A., Stopa, M., Lobacz, M., Niemczyk, M., Rutowska, K., Radko, A., Mikunda, A., & Czarnecka, D. (2017). Influence of energy drinks on hemodynamic parameters-randomized double-blind placebo controlled cross-over study [Conference Abstract]. *European Heart Journal*, 38, 318. | Follow up period too short to show results on cardiovascular disease |
| Yang, Y. Z., Chen, R. Z., & Zhang, J. N. (2001). [Observation on collaborative treatment of dilated cardiomyopathy]. *Zhongguo Zhong Xi Yi Jie He Za Zhi*, 21(4), 254-256. | Herbal treatment with undocumented active compounds |
| Liu, D., Lu, Z., Sun, Q., Wang, B., & Zhu, Z. (2018). Taurine supplementation improves vascular function in prehypertension: a randomized, double-blind, placebo-controlled study [Journal article; Conference proceeding]. *Journal of Hypertension*, 36, e145. | Poster abstract only, no available data of pre- and post-intervention endpoint |
| Ahmadian, M., Roshan, V. D., Aslani, E., & Stannard, S. R. (2017). Taurine supplementation has anti-atherogenic and anti-inflammatory effects before and after incremental exercise in heart failure. *Ther Adv Cardiovasc Dis*, 11(7), 185-194. | Studies devoid of outcomes of interest |
| Arrieta, F., Balsa, J. A., de la Puerta, C., Botella, J. I., Zamarron, I., Elias, E., del Rio, J. I. P., Alonso, P., Candela, A., Blanco-Colio, L. M., Egido, J., Navarro, P., & Vazquez, C. (2014). Phase IV Prospective Clinical Study to Evaluate the Effect of Taurine on Liver Function in Postsurgical Adult Patients Requiring Parenteral Nutrition. *Nutrition in Clinical Practice*, 29(5), 672-680. | Studies devoid of outcomes of interest |
| Batitucci, G., Brandao, C. F. C., De Carvalho, F. G., Marchini, J. S., Pfrimer, K., Ferrioli, E., Cunha, F. Q., Papoti, M., Terrazas, S., Junqueira-Franco, M. V. M., da Silva, A. S. R., & Freitas, E. C. (2019). Taurine supplementation increases irisin levels after high intensity physical training in obese women. *Cytokine,* 123, 154741. | Studies devoid of outcomes of interest |
| Franconi, F., Bennardini, F., Mattana, A., Miceli, M., Ciuti, M., Mian, M., Gironi, A., Anichini, R., & Seghieri, G. (1995). Plasma and platelet taurine are reduced in subjects with insulin-dependent diabetes-mellitus - effects of taurine supplementation. *American Journal of Clinical Nutrition*, 61(5), 1115-1119. | Studies devoid of outcomes of interest |
| Fujita, T., Ando, K., Noda, H., Ito, Y., & Sato, Y. (1987). Effects of increased adrenomedullary activity and taurine in young patients with borderline hypertension. *Circulation*, 75(3), 525-532 | Studies devoid of outcomes of interest |
| Gordeev, I. G., Pokrovskaya, E. M., & Luchinkina, E. E. (2012). Taurine effects on the occurrence of cardiac arrhythmias and QT interval dispersion in patients with post-infarction cardiosclerosis and chronic heart failure: a comparative randomised study. *Cardiovascular Therapy and Prevention,* 11(1), 63-68. | Studies devoid of outcomes of interest |
| Jeejeebhoy, F., Keith, M., Freeman, M., Barr, A., McCall, M., Kurian, R., Mazer, D., & Errett, L. (2002). Nutritional supplementation with MyoVive repletes essential cardiac myocyte nutrients and reduces left ventricular size in patients with left ventricular dysfunction [Article]. *American Heart Journal*, 143(6), 1092-1100. | Studies devoid of outcomes of interest |
| Moloney, M. A., Casey, R. G., O'Donnell, D. H., Fitzgerald, P., Thompson, C., & Bouchier-Hayes, D. J. (2010). Two weeks taurine supplementation reverses endothelial dysfunction in young male type 1 diabetics [Article]. *Diabetes and Vascular Disease Research*, 7(4), 300-310. | Studies devoid of outcomes of interest |
| Roshan, V. D., Khalafi, M. K., & Choobineh, S. (2011). Effects of taurine supplementation on response of the cardiac injury biomarkers to bruce diagnostic protocol in patients with heart failure [Article]. *Koomesh*, 13(1), 73-82. | Studies devoid of outcomes of interest |
| Singh, R. B., Kartikey, K., Charu, A. S., Niaz, M. A., & Schaffer, S. (2003). Effect of taurine and coenzyme Q10 in patients with acute myocardial infarction. *Adv Exp Med Biol*, 526, 41-48. | Studies devoid of outcomes of interest |
| Van Hove, J. L. K., Freehauf, C. L., Ficicioglu, C., Pena, L. D. M., Moreau, K. L., Henthorn, T. K., Christians, U., Jiang, H., Cowan, T. M., Young, S. P., Hite, M., Friederich, M. W., Stabler, S. P., Spector, E. B., Kronquist, K. E., Thomas, J. A., Emmett, P., Harrington, M. J., Pyle, L., . . . MacLean, K. N. (2019). Biomarkers of oxidative stress, inflammation, and vascular dysfunction in inherited cystathionine -synthase deficient homocystinuria and the impact of taurine treatment in a phase 1/2 human clinical trial. *Journal of Inherited Metabolic Disease*, 42(3), 424-437. | Studies devoid of outcomes of interest |
| Zhang, M., Bi, L. F., Fang, J. H., Su, X. L., Da, G. L., Kuwamori, T., & Kagamimori, S. (2004). Beneficial effects of taurine on serum lipids in overweight or obese non-diabetic subjects [Article]. *Amino Acids*, 26(3), 267-271. | Studies devoid of outcomes of interest |

**Table S4. Assessment of evidence quality for each outcome**

| **Quality assessment** | | | | | | | **No of patients** | | **Effect** | | **Quality** | **Importance** |
| --- | --- | --- | --- | --- | --- | --- | --- | --- | --- | --- | --- | --- |
|  |  |  |  |  |  |  |  |  |  |  |  |  |
| **No of studies** | **Design** | **Risk of bias** | **Inconsistency** | **Indirectness** | **Imprecision** | **Other considerations** | **Taurine /**  **Placebo** | | **Relative**  **(95% CI)** | **Absolute** |  |  |
| **Heart rate overall** | | | | | | | | | | | | |
| 9 | randomised trials | No serious bias | no serious inconsistency | no serious indirectness | no serious imprecision | none | 133 | 132 | WMD: -3.579 bpm lower, (-6.044 to -1.114 lower) |  | HIGH | CRITICAL |
| **Heart rate subgroup analysis (Diabetes)** | | | | | | | | | | | | |
| 1 | randomised trials | No serious bias | no serious inconsistency | no serious indirectness | no serious imprecision | none | 19 | 21 | WMD: 0.000 bpm, (-2.556 to 2.556) |  | HIGH | CRITICAL |
| **Heart rate subgroup analysis (Healthy)** | | | | | | | | | | | | |
| 1 | randomised trials | No serious bias | no serious inconsistency | no serious indirectness | no serious imprecision | none | 15 | 14 | WMD: -1.700 bpm lower, (-2.978 to -0.422 lower) |  | HIGH | CRITICAL |
| **Heart rate subgroup analysis (Heart failure)** | | | | | | | | | | | | |
| 5 | randomised trials | No serious bias | no serious inconsistency | no serious indirectness | no serious imprecision | none | 70 | 69 | WMD: -3.898 bpm lower, ( -4.679 to -3.116 lower) |  | HIGH | CRITICAL |
| **Heart rate subgroup analysis (Other disease)** | | | | | | | | | | | | |
| 2 | randomised trials | No serious bias | no serious inconsistency | no serious indirectness | no serious imprecision | none | 29 | 28 | WMD: -6.197 bpm lower, (-15.248 to 2.853 lower) |  | HIGH | CRITICAL |
| **Systolic blood pressure overall** | | | | | | | | | | | | |
| 12 | randomised trials | No serious bias | no serious inconsistency | no serious indirectness | no serious imprecision | none | 259 | 259 | WMD: -3.999 mm Hg lower, (-7.293 to -0.706 lower) |  | HIGH | CRITICAL |
| **Systolic blood pressure subgroup analysis (Diabetes)** | | | | | | | | | | | | |
| 4 | randomised trials | No serious bias | no serious inconsistency | no serious indirectness | no serious imprecision | none | 108 | 111 | WMD: 0.061 mm Hg higher, (-2.001 to 2.123) |  | HIGH | CRITICAL |
| **Systolic blood pressure subgroup analysis (Healthy)** | | | | | | | | | | | | |
| 1 | randomised trials | No serious bias | no serious inconsistency | no serious indirectness | no serious imprecision | none | 15 | 14 | WMD: -3.400 mm Hg lower, (-4.892 to -1.908 lower) |  | HIGH | CRITICAL |
| **Systolic blood pressure subgroup analysis (Health failure)** | | | | | | | | | | | | |
| 4 | randomised trials | No serious bias | no serious inconsistency | no serious indirectness | no serious imprecision | none | 63 | 62 | WMD: -9.817 mm Hg lower, (-18.575 to -1.060 lower) |  | HIGH | CRITICAL |
| **Systolic blood pressure subgroup analysis (Hypertension)** | | | | | | | | | | | | |
| 2 | randomised trials | No serious bias | no serious inconsistency | no serious indirectness | no serious imprecision | none | 53 | 52 | WMD: -9.457 mm Hg lower, (-18.963 to 0.049 lower) |  | HIGH | CRITICAL |
| **Systolic blood pressure subgroup analysis (Other disease)** | | | | | | | | | | | | |
| 1 | randomised trials | No serious bias | no serious inconsistency | no serious indirectness | no serious imprecision | none | 20 | 20 | WMD: 4.600 mm Hg higher, (1.555 to 7.645 higher) |  | HIGH | IMPORTANT |
| **Diastolic blood pressure overall** | | | | | | | | | | | | |
| 12 | randomised trials | No serious bias | no serious inconsistency | no serious indirectness | no serious imprecision | none | 259 | 259 | WMD: -3.137 mm Hg lower, (-4.865 to -1.408 lower) |  | HIGH | CRITICAL |
| **Diastolic blood pressure subgroup analysis (Diabetes)** | | | | | | | | | | | | |
| 4 | randomised trials | No serious bias | no serious inconsistency | no serious indirectness | no serious imprecision | none | 108 | 111 | WMD: -0.132 mm Hg lower, (-1.990 to 1.726) |  | HIGH | CRITICAL |
| **Diastolic blood pressure subgroup analysis (Healthy)** | | | | | | | | | | | | |
| 1 | randomised trials | No serious bias | no serious inconsistency | no serious indirectness | no serious imprecision | none | 15 | 14 | WMD: -0.900 mm Hg lower, (-2.141 to 0.341) |  | HIGH | CRITICAL |
| **Diastolic blood pressure subgroup analysis (Heart failure)** | | | | | | | | | | | | |
| 4 | randomised trials | No serious bias | no serious inconsistency | no serious indirectness | no serious imprecision | none | 63 | 62 | WMD: -3.758 mm Hg lower, (-7.680 to 0.165) |  | HIGH | CRITICAL |
| **Diastolic blood pressure subgroup analysis (Hypertension)** | | | | | | | | | | | | |
| 2 | randomised trials | No serious bias | no serious inconsistency | no serious indirectness | no serious imprecision | none | 53 | 52 | WMD: -3.137 mm Hg lower, (-4.865 to -1.408) |  | HIGH | CRITICAL |
| **Diastolic blood pressure subgroup analysis (Other disease)** | | | | | | | | | | | | |
| 1 | randomised trials | No serious bias | no serious inconsistency | no serious indirectness | no serious imprecision | none | 20 | 20 | WMD: -0.250 mm Hg lower, (-2.603 to 2.103) |  | HIGH | CRITICAL |
| **Left ventricular ejection fraction overall** | | | | | | | | | | | | |
| 6 | randomised trials | No serious bias | no serious inconsistency | no serious indirectness | no serious imprecision | none | 106 | 105 | WMD: 4.981 %, higher, (1.556 to 8.407 higher) |  | HIGH | CRITICAL |
| **Left ventricular ejection fraction subgroup analysis (Heart failure)** | | | | | | | | | | | | |
| 3 | randomised trials | No serious bias | no serious inconsistency | no serious indirectness | no serious imprecision | none | 40 | 39 | WMD: 5.370 %, higher, (2.982 to 7.757 higher) |  | HIGH | CRITICAL |
| **Left ventricular ejection fraction subgroup analysis (Other disease)** | | | | | | | | | | | | |
| 3 | randomised trials | No serious bias | no serious inconsistency | no serious indirectness | no serious imprecision | none | 66 | 66 | WMD: 4.609, higher, (-3.510 to 12.728) |  | HIGH | CRITICAL |
| **New York Heart Association Functional Classification overall** | | | | | | | | | | | | |
| 6 | randomised trials | No serious bias | no serious inconsistency | no serious indirectness | no serious imprecision | none | 120 | 120 | WMD: -0.403 lower, (-0.522 to -0.283 lower) |  | HIGH | CRITICAL |
| **New York Heart Association Functional Classification subgroup analysis (Heart failure)** | | | | | | | | | | | | |
| 3 | randomised trials | No serious bias | no serious inconsistency | no serious indirectness | no serious imprecision | none | 54 | 54 | WMD: -0.356 lower, (-0.484 to -0.227 lower) |  | HIGH | CRITICAL |
| **New York Heart Association Functional Classification subgroup analysis (Other disease)** | | | | | | | | | | | | |
| 3 | randomised trials | No serious bias | no serious inconsistency | no serious indirectness | no serious imprecision | none | 66 | 66 | WMD: -0.383 lower, (-0.680 to -0.085 lower) |  | HIGH | CRITICAL |
| Adverse Events | | | | | | | | | | | | |
| 20 | randomised trials | No serious bias | no serious inconsistency | no serious indirectness | no serious imprecision | none |  |  | Odds Ratio: 1.328 higher, (0.663 to 2.663) |  | HIGH | CRITICAL |

CI, confidence interval; WMD, weighted mean difference;

**Figure S1.** Summary of quality assessment of studies included in the meta-analysis using Cochrane risk of bias 2 tool

**
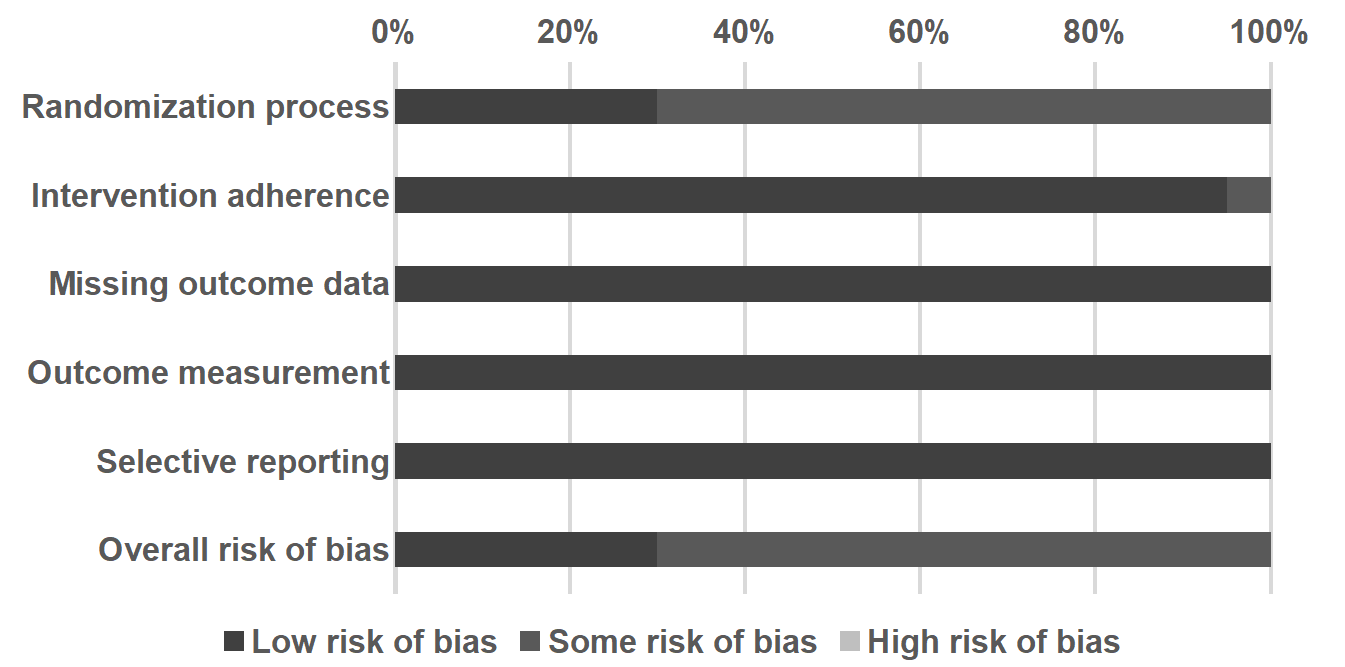
**

**Figure S2.** Results of sensitivity analysis using the one-study removal method to assess the impact of taurine on the overall effect size for heart rate


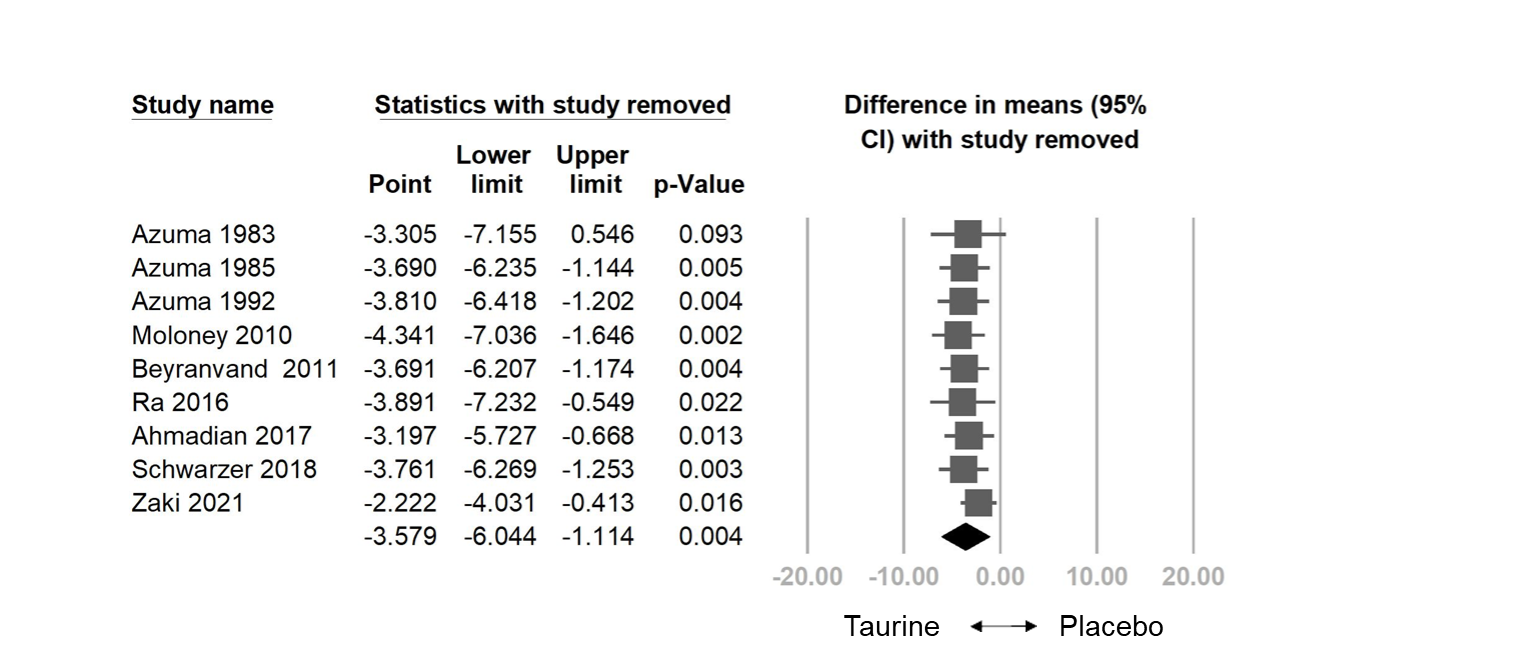


The omission of study 4 (Moloney, 2010) or 8 (Schwarzer, 2018) seems to have a relatively larger influence (when compared with other studies) on the estimation of the overall effect size. Omitting study 4 causes the weighted mean difference to decrease by roughly 0.8, whereas omitting study 8 causes the weighted mean difference to increase by roughly 1.3.

**Figure S3.** Meta-regression analysis showing the relationship between the total taurine dose throughout the treatment periods and heart rate


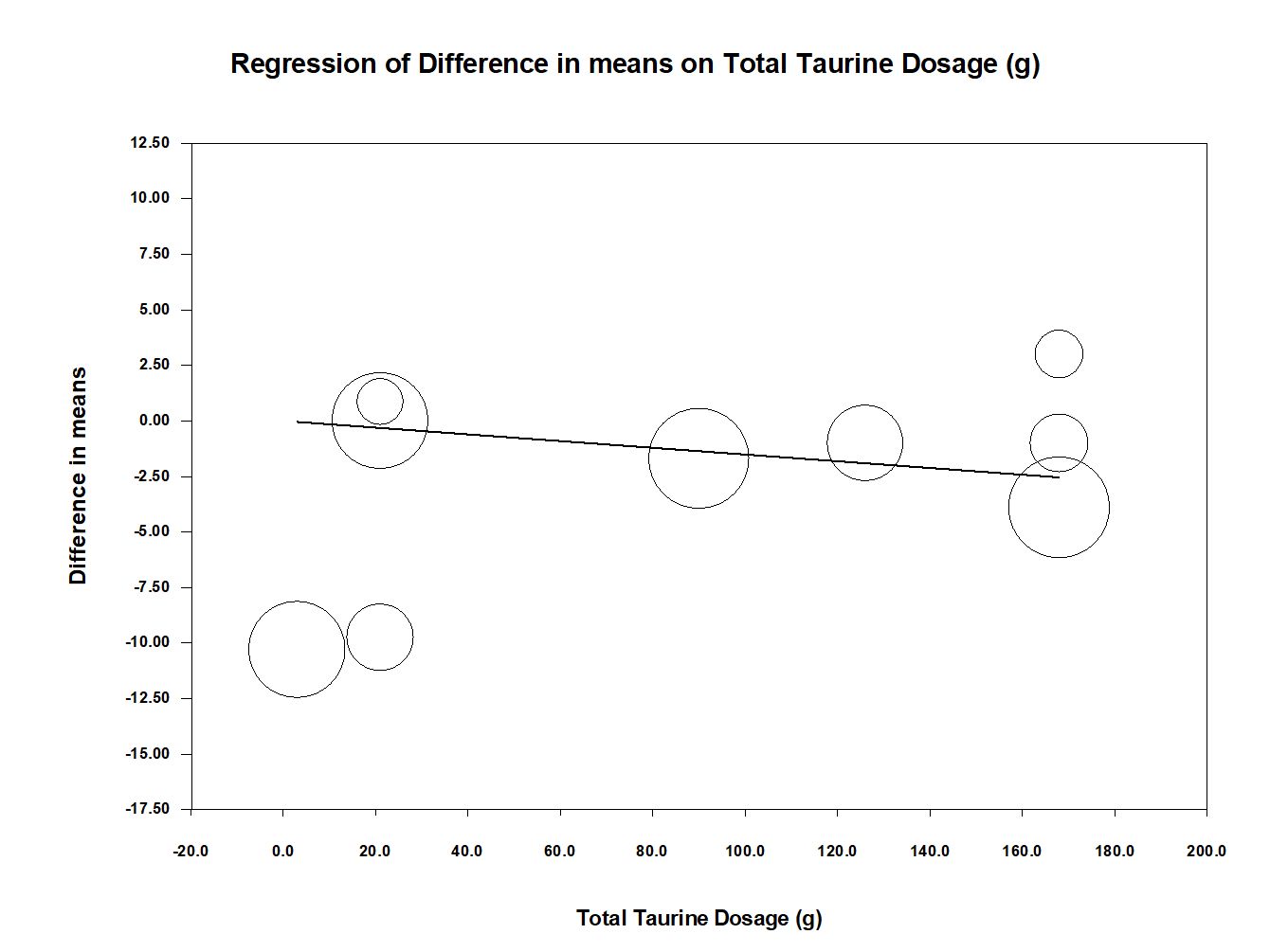


**Figure S4.** Results of subgroup analysis to investigate the effect of taurine on heart rate in diabetes, healthy, heart failure, and other disease populations.


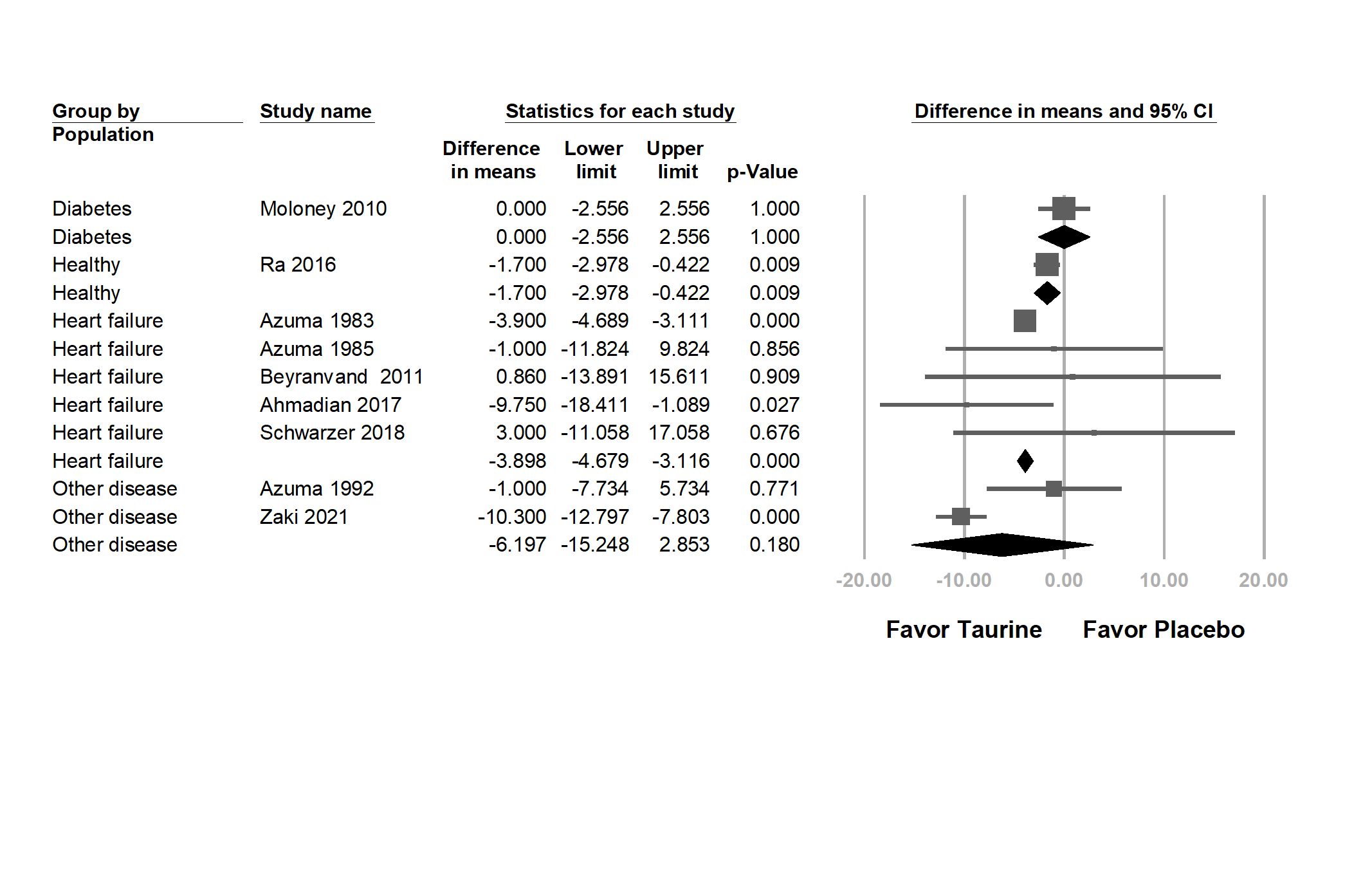


**Figure S5.** Results of sensitivity analysis using the one-study removal method to assess the impact of taurine on the overall effect size for (A) systolic blood pressure (B) diastolic blood pressure.


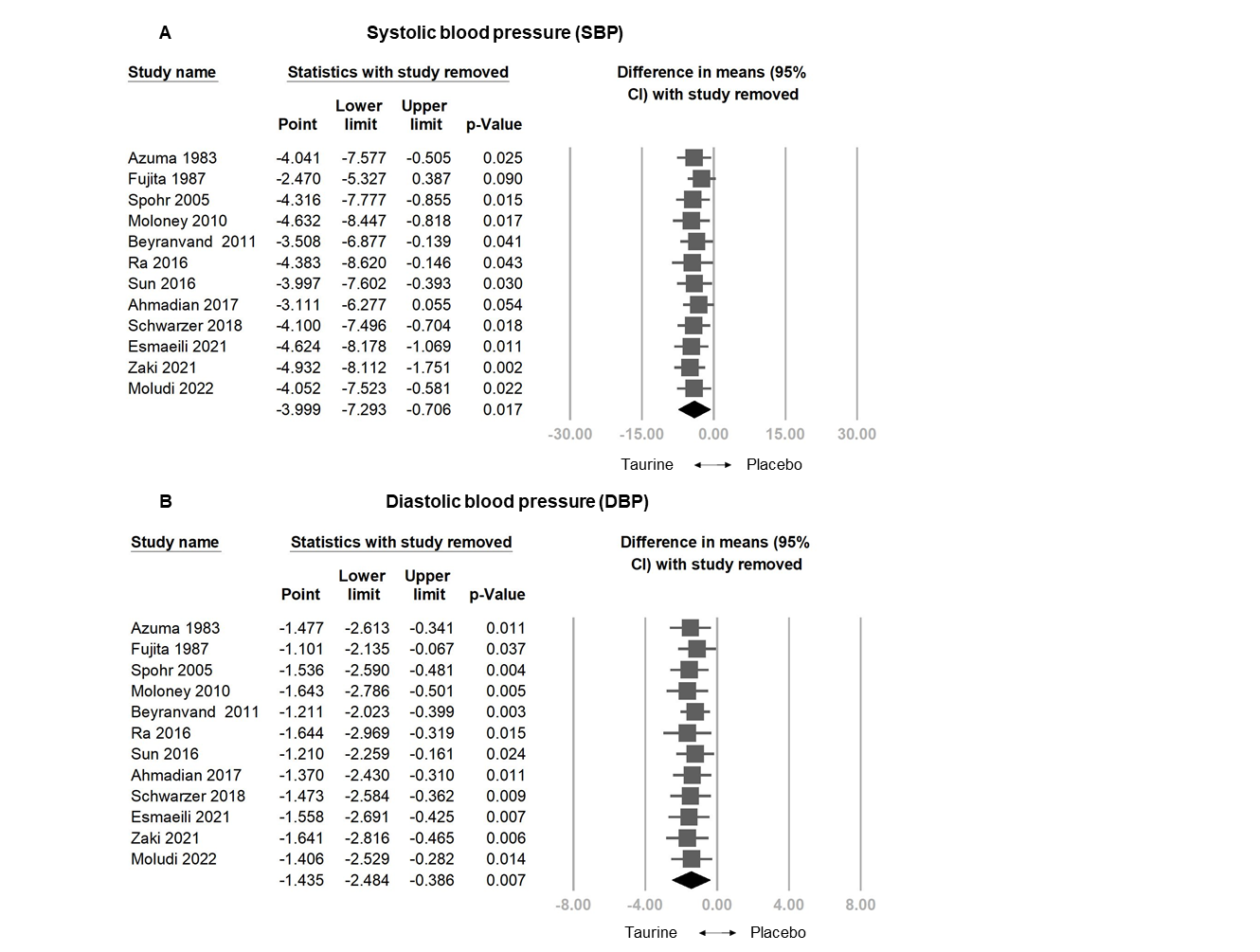


(A)The omission of study 2 (Fujita, 1987) or 11 (Zaki, 2021) seems to have a relatively larger influence (when compared with other studies) on the estimation of the overall effect size. Omitting study 2 causes the weighted mean difference to increase by roughly 1.6, whereas omitting study 11 causes the weighted mean difference to increase by roughly 0.9.

(B)The omission of study 2 (Fujita, 1987) or 6 (Ra, 2016) seems to have a relatively larger influence (when compared with other studies) on the estimation of the overall effect size. Omitting study 2 causes the weighted mean difference to increase by roughly 0.3, whereas omitting study 6 causes the weighted mean difference to decrease by roughly 0.3.

**Figure S6.** Meta-regression analysis showing the relationship between the total taurine dose throughout the treatment periods and (A) systolic blood pressure (B) diastolic blood pressure

**
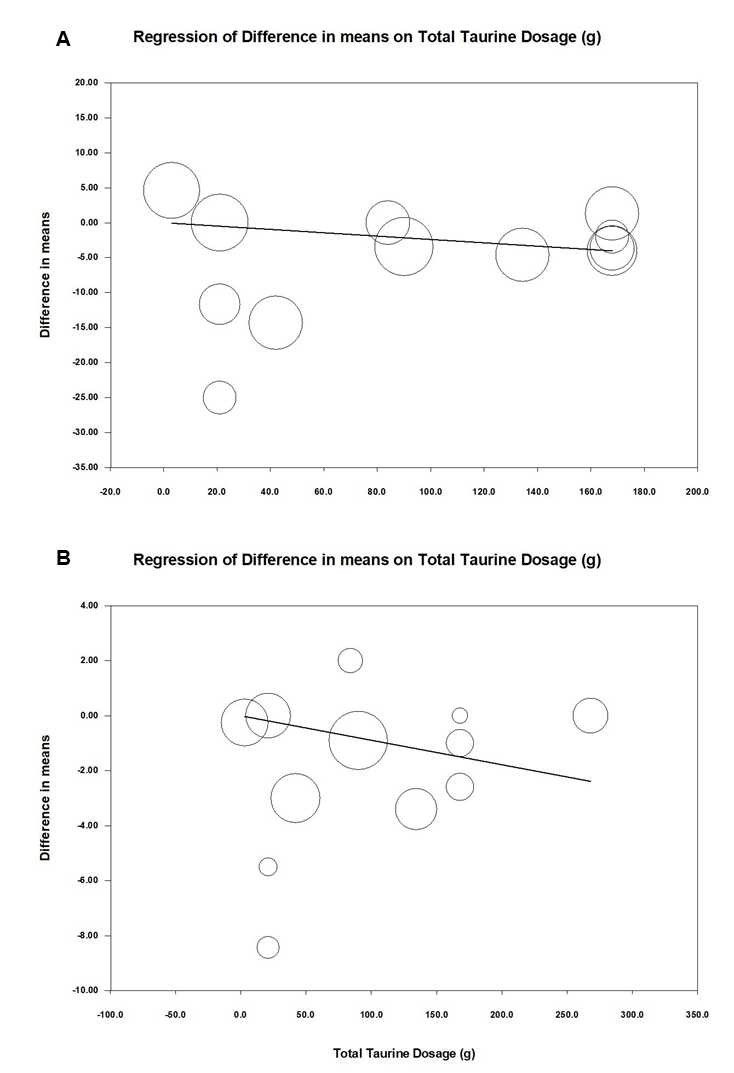
**

**Figure S7.** Results of subgroup analysis to investigate the effect of taurine on systolic blood pressure in diabetes, healthy, heart failure, hypertension, and other disease populations.


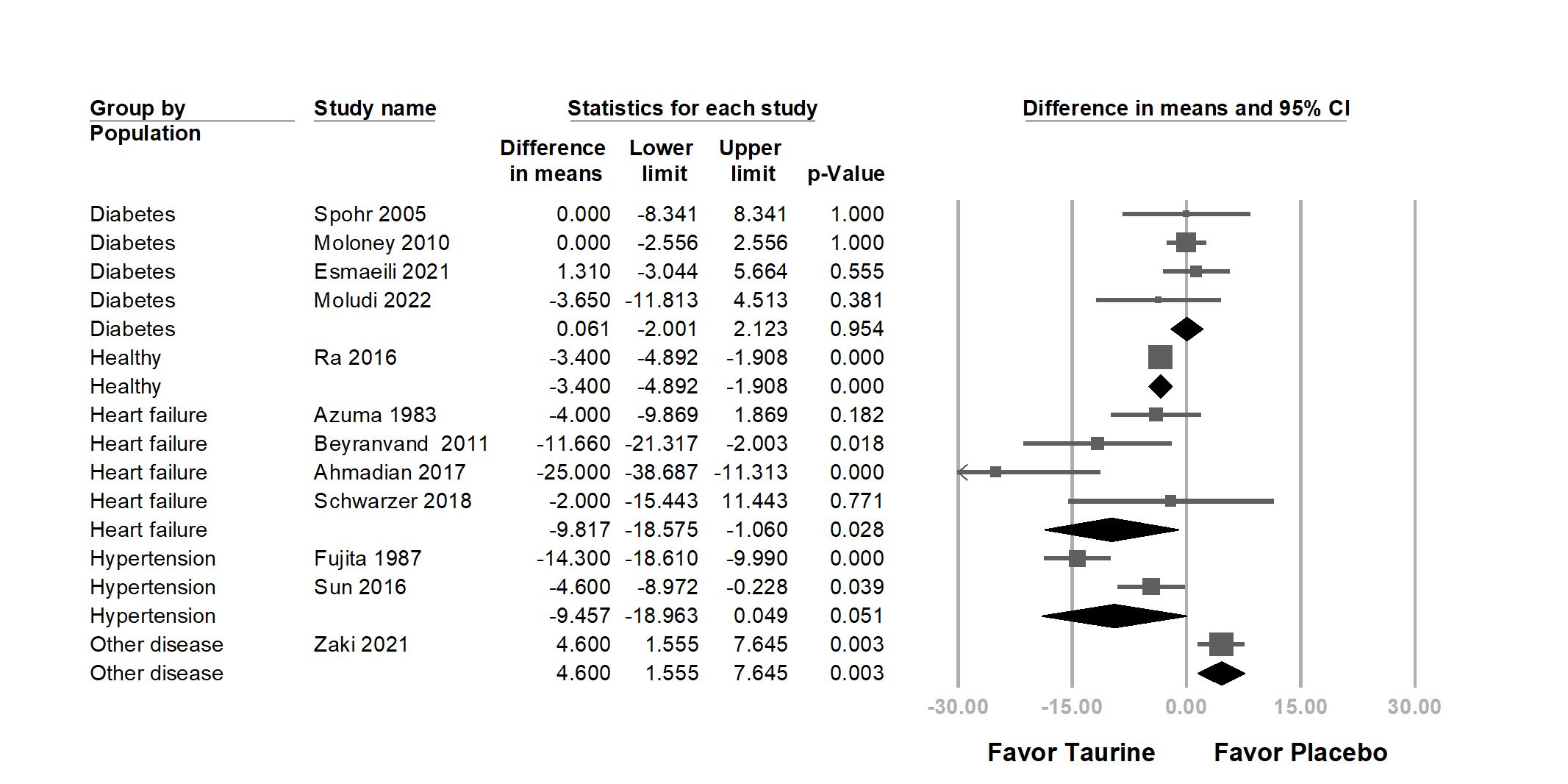


**Figure S8.** Results of subgroup analysis to investigate the effect of taurine on diastolic blood pressure in diabetes, healthy, heart failure, and other disease populations.

**
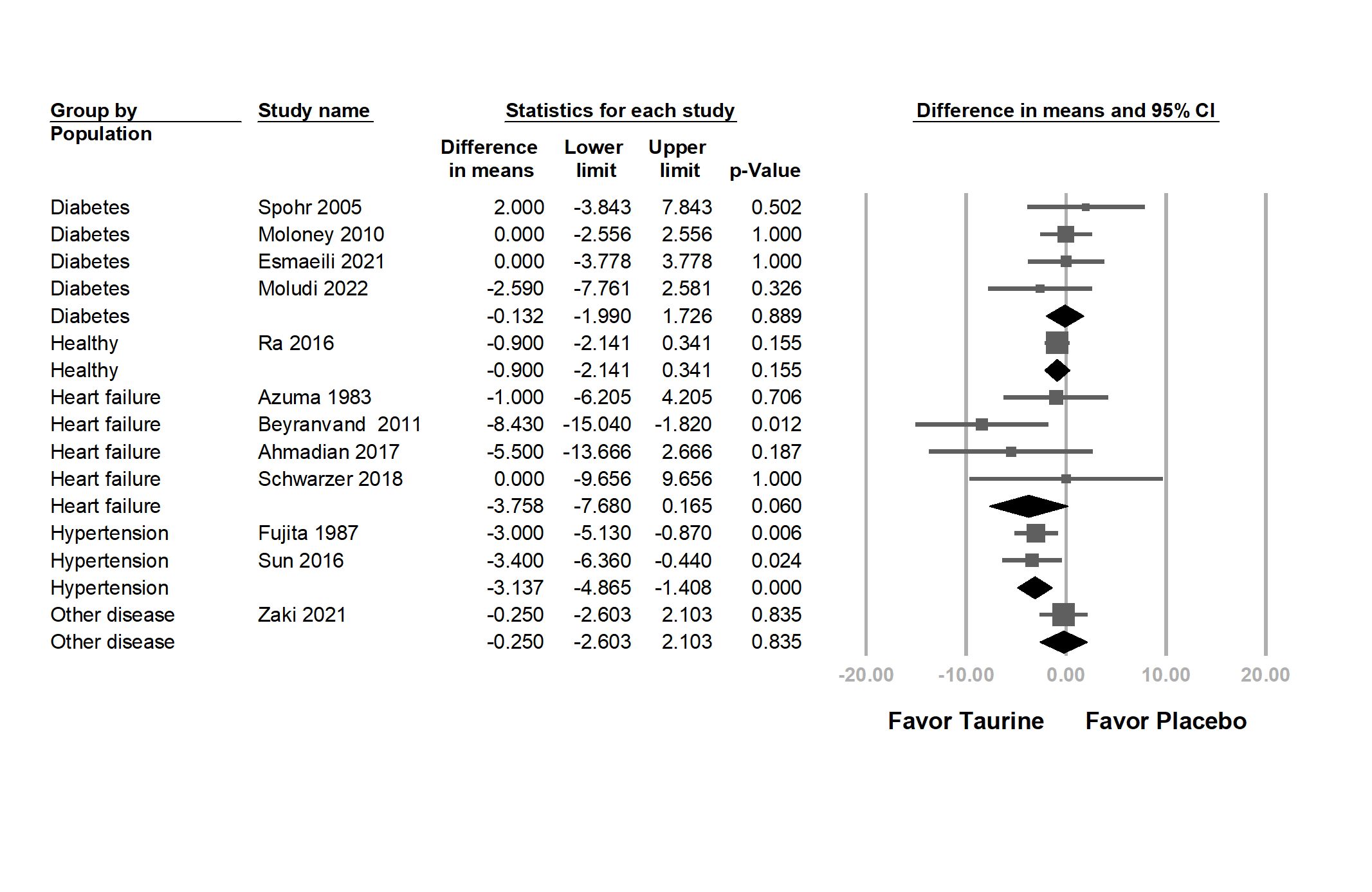
**

**Figure S9.** Results of sensitivity analysis using the one-study removal method to assess the impact of taurine on the overall effect size for left ventricular ejection fraction

**
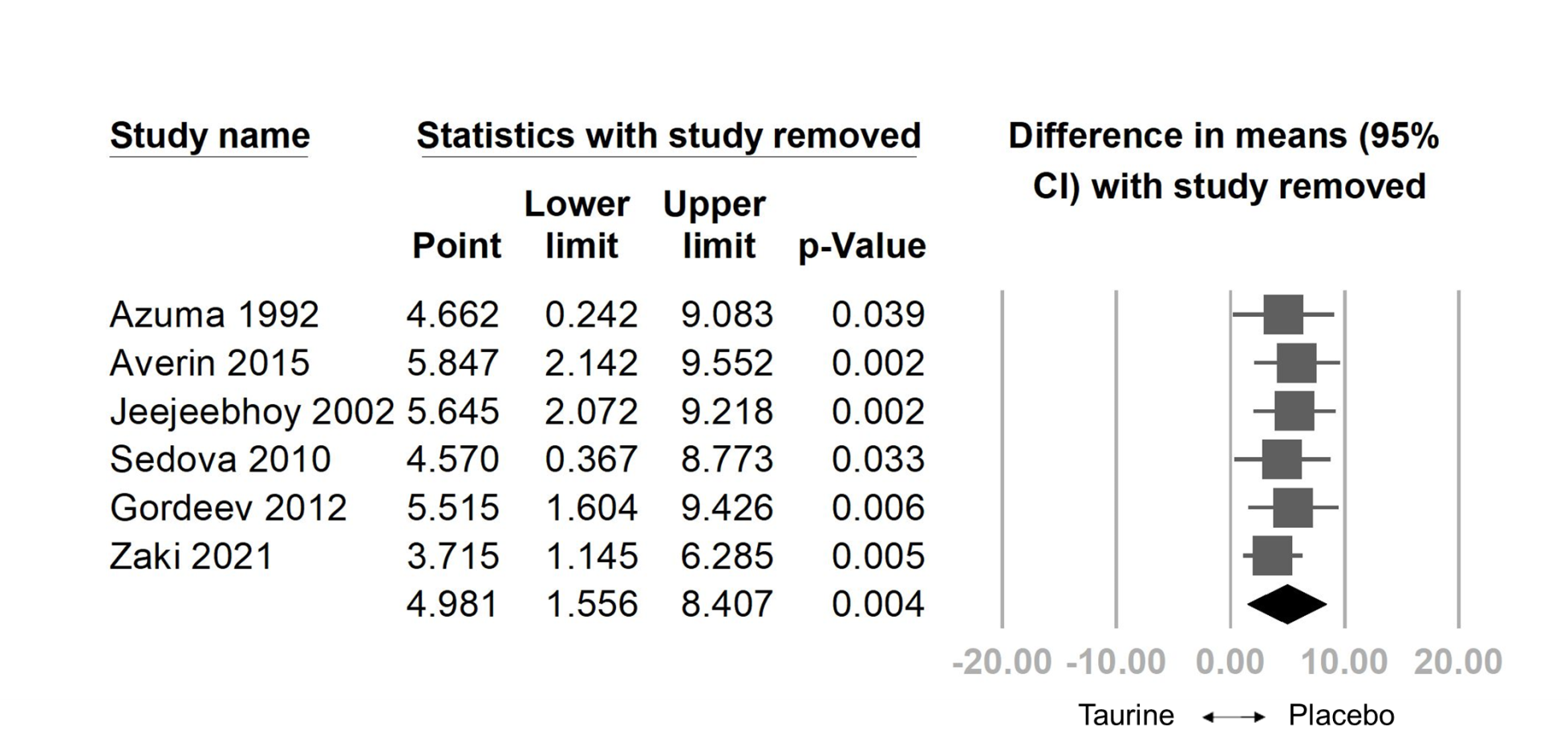
**

The omission of study 2 (Averin, 2015) or 6 (Zaki, 2021) seems to have a relatively larger influence (when compared with other studies) on the estimation of the overall effect size. Omitting study 2 causes the weighted mean difference to increase by roughly 0.8, whereas omitting study 6 causes the weighted mean difference to decrease by roughly 1.3.

**Figure S10.** Meta-regression analysis showing the relationship between the total taurine dose throughout the treatment periods and left ventricular ejection fraction


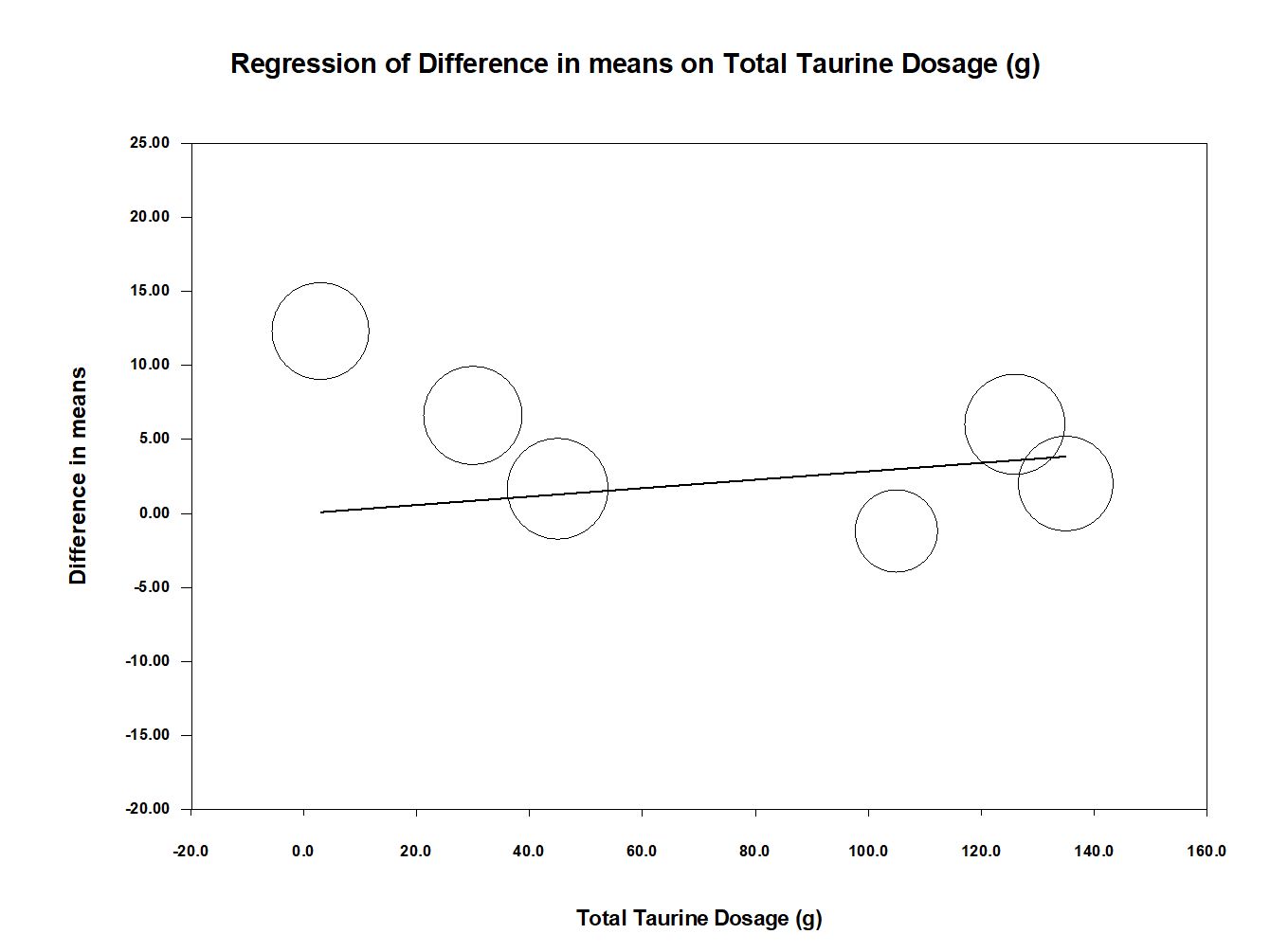


**Figure S11.** Results of subgroup analysis to investigate the effect of taurine on left ventricular ejection fraction in heart failure and other disease populations.

**
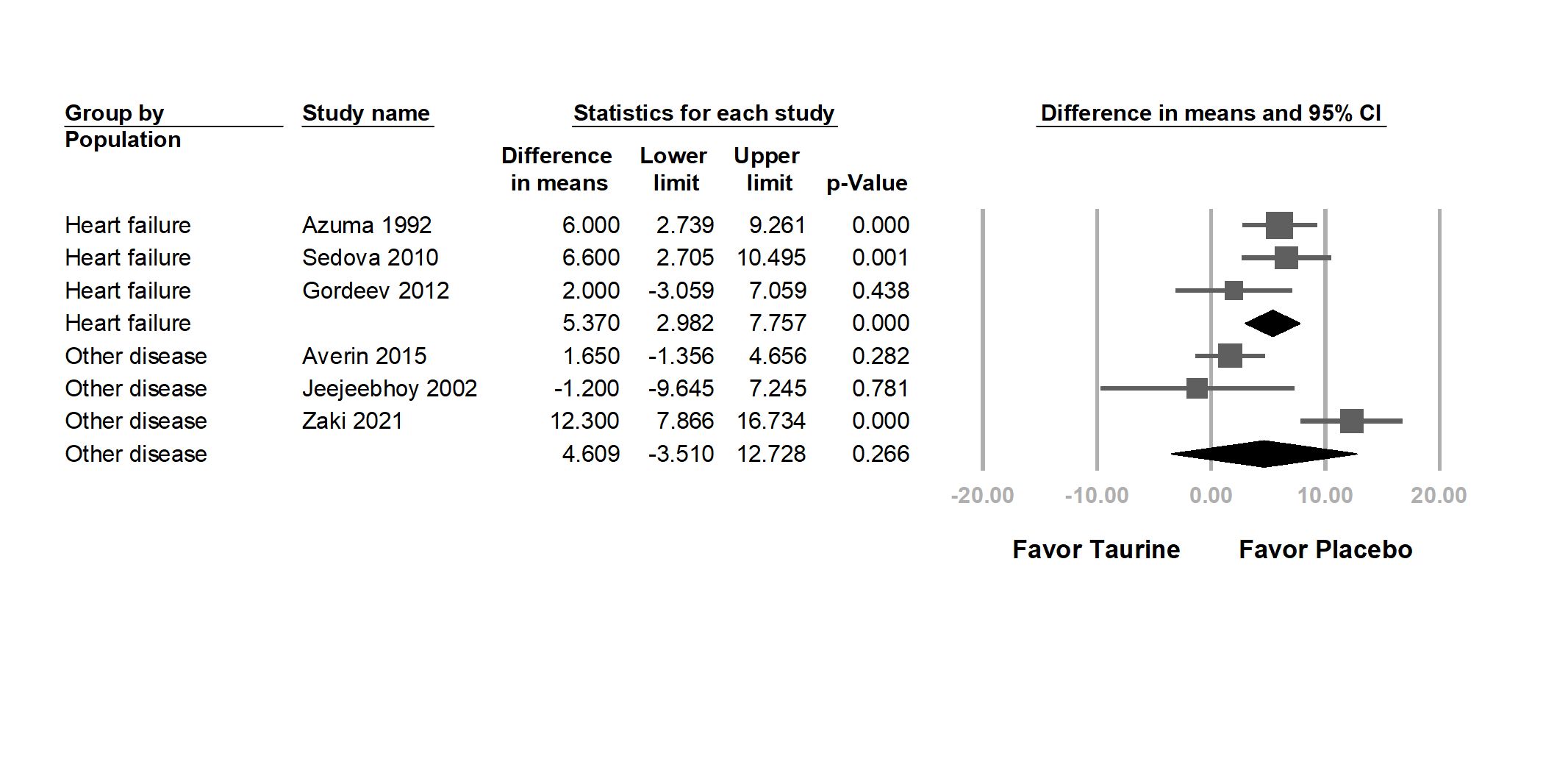
**

**Figure S12.** Results of sensitivity analysis using the one-study removal method to assess the impact of taurine on the overall effect size for New York Heart Association Functional Classification

**
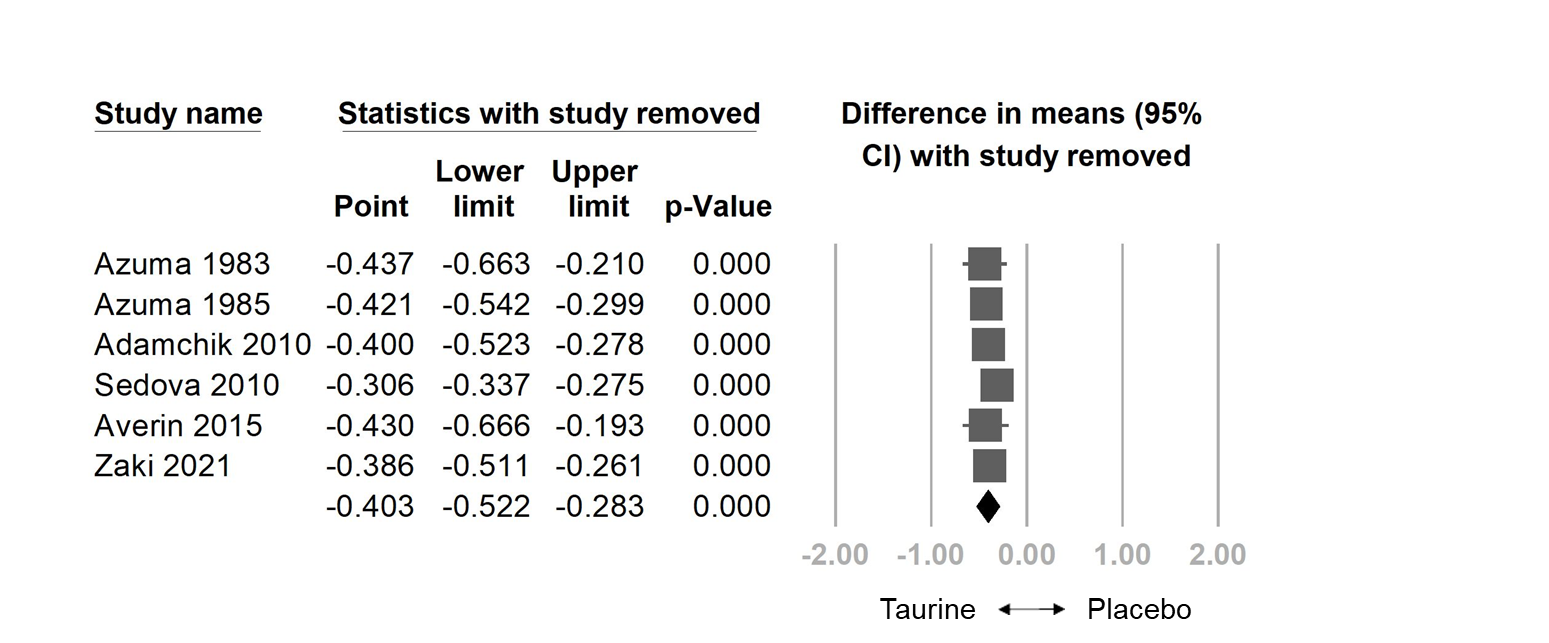
**

The omission of study 1 (Azuma, 1983) or 4 (Sedova, 2010) seems to have a relatively larger influence (when compared with other studies) on the estimation of the overall effect size. Omitting study 1 causes the weighted mean difference to decrease by roughly 0.3, whereas omitting study 4 causes the weighted mean difference to increase by roughly 1.0.

**Figure S13.** Meta-regression analysis showing the relationship between the total taurine dose throughout the treatment periods and New York Heart Association Functional Classification


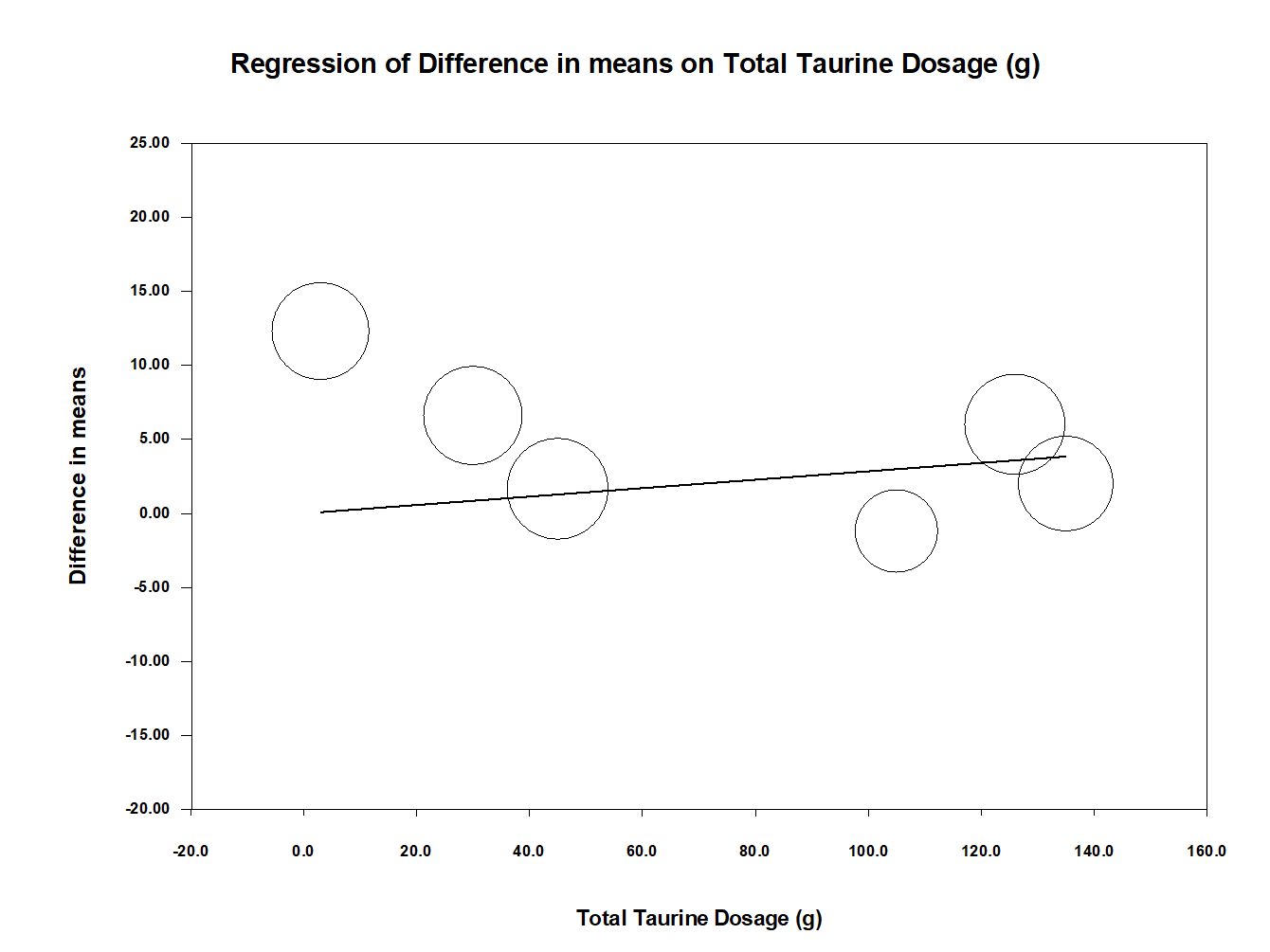


**Figure S14.** Results of subgroup analysis to investigate the effect of taurine on New York Heart Association Functional Classification in heart failure and other disease populations.

**
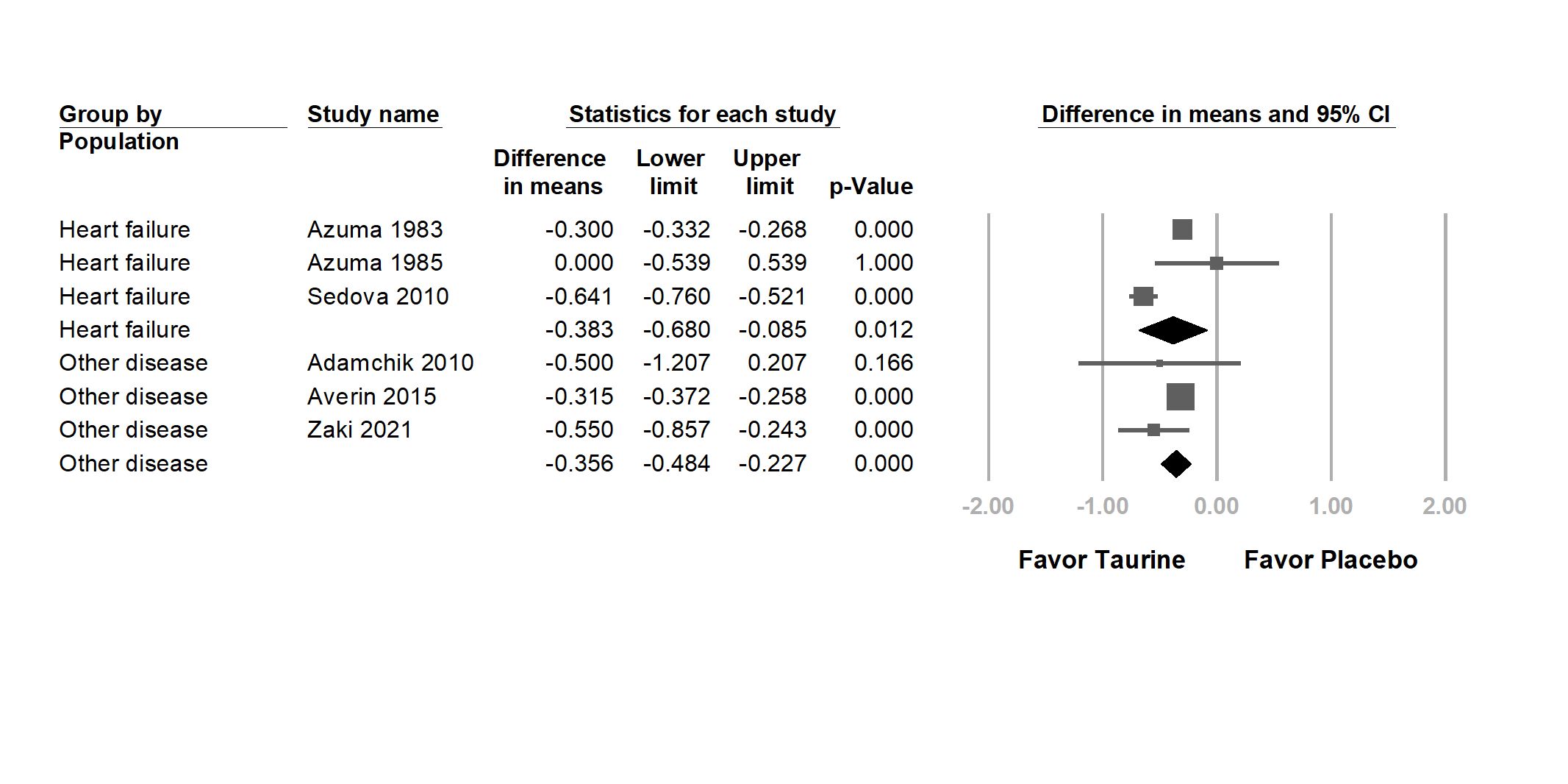
**

**Figure S15**. Funnel plot depicting the distribution of effect sizes for heart rate across studies


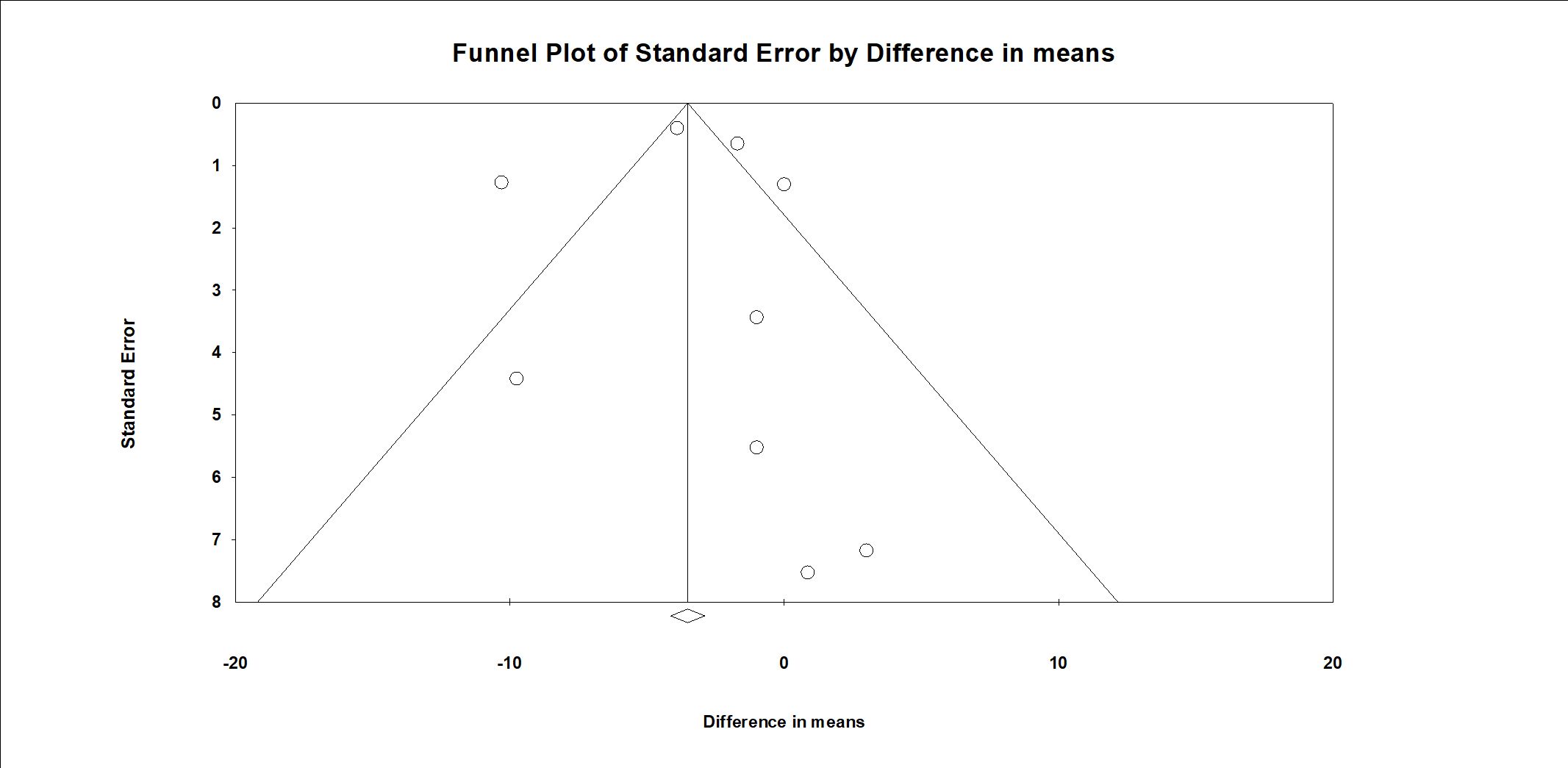


**Figure S16**. Funnel plot depicting the distribution of effect sizes for (A) systolic blood pressure (B) diastolic blood pressure across studies


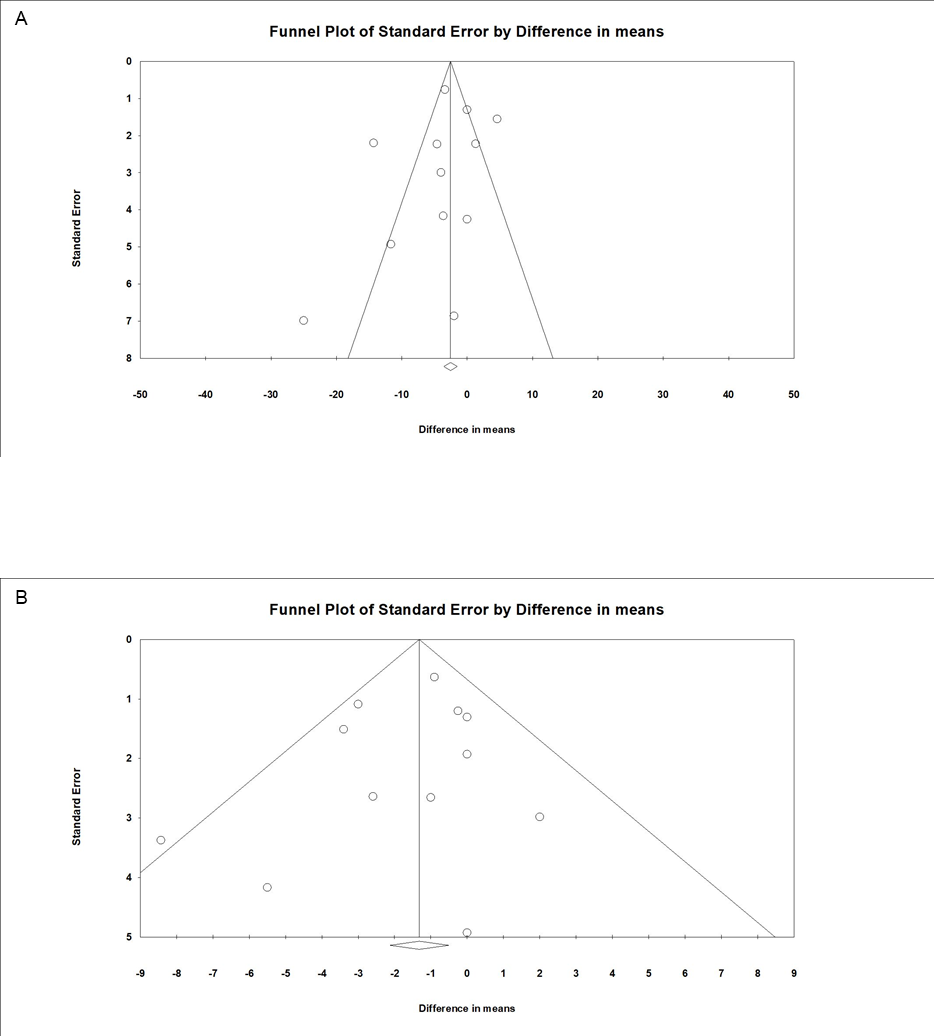


**Figure S17**. Funnel plot depicting the distribution of effect sizes for left ventricular ejection fraction across studies


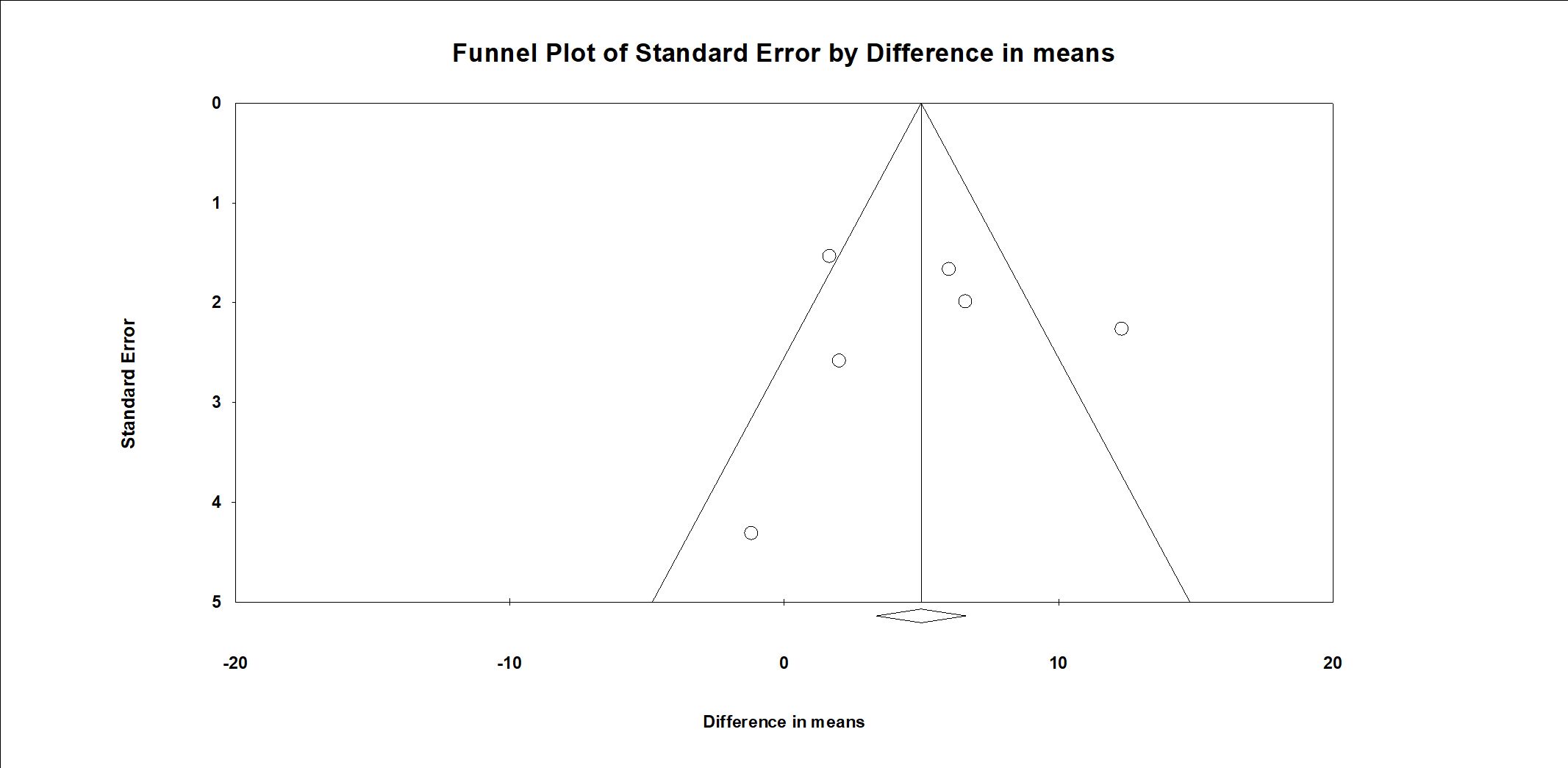


**Figure S18**. Funnel plot depicting the distribution of effect sizes for New York Heart Association Functional Classification across studies


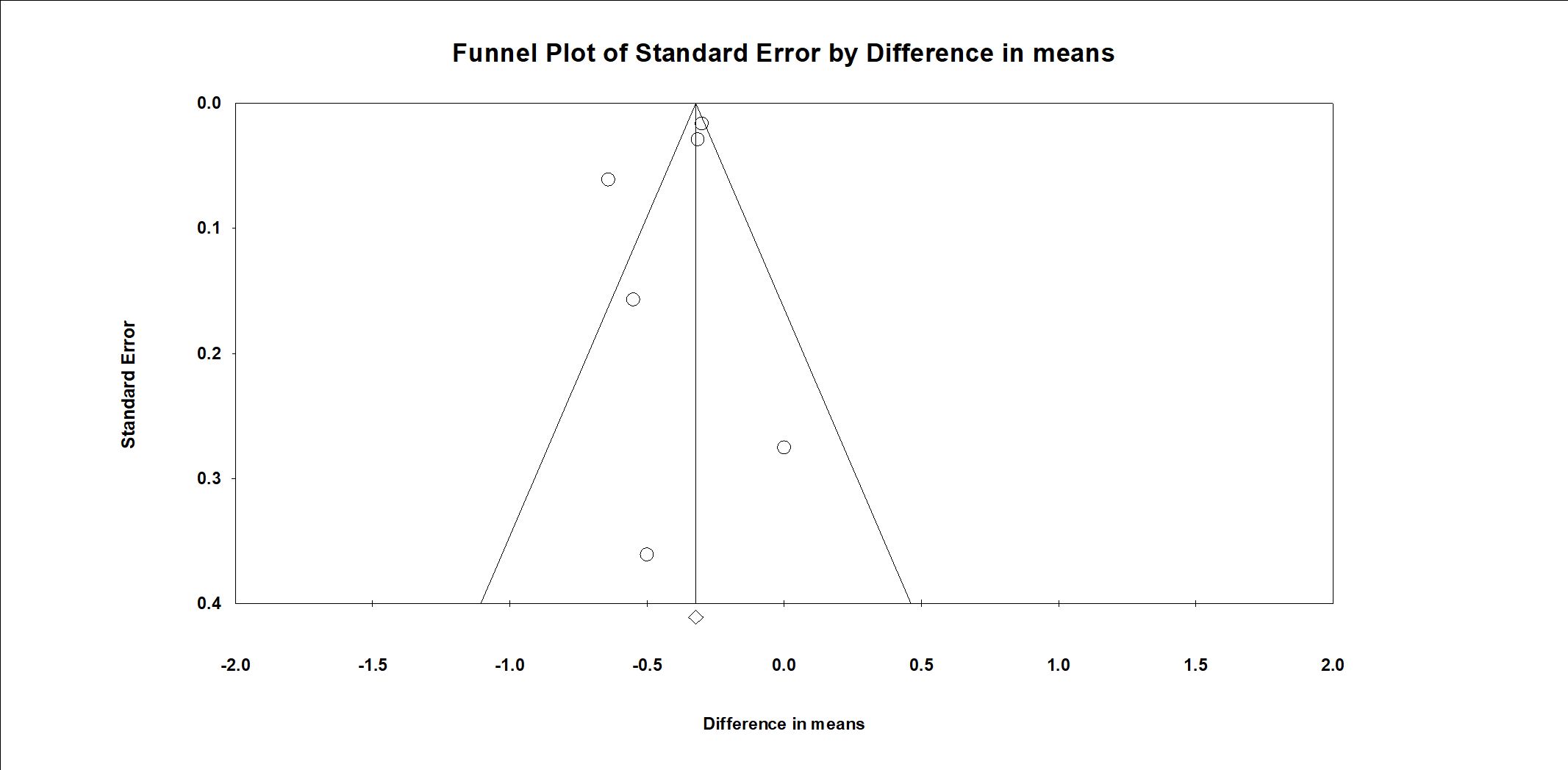


**Figure S19.** Forest plot of the treatment-associated adverse effect rates

**
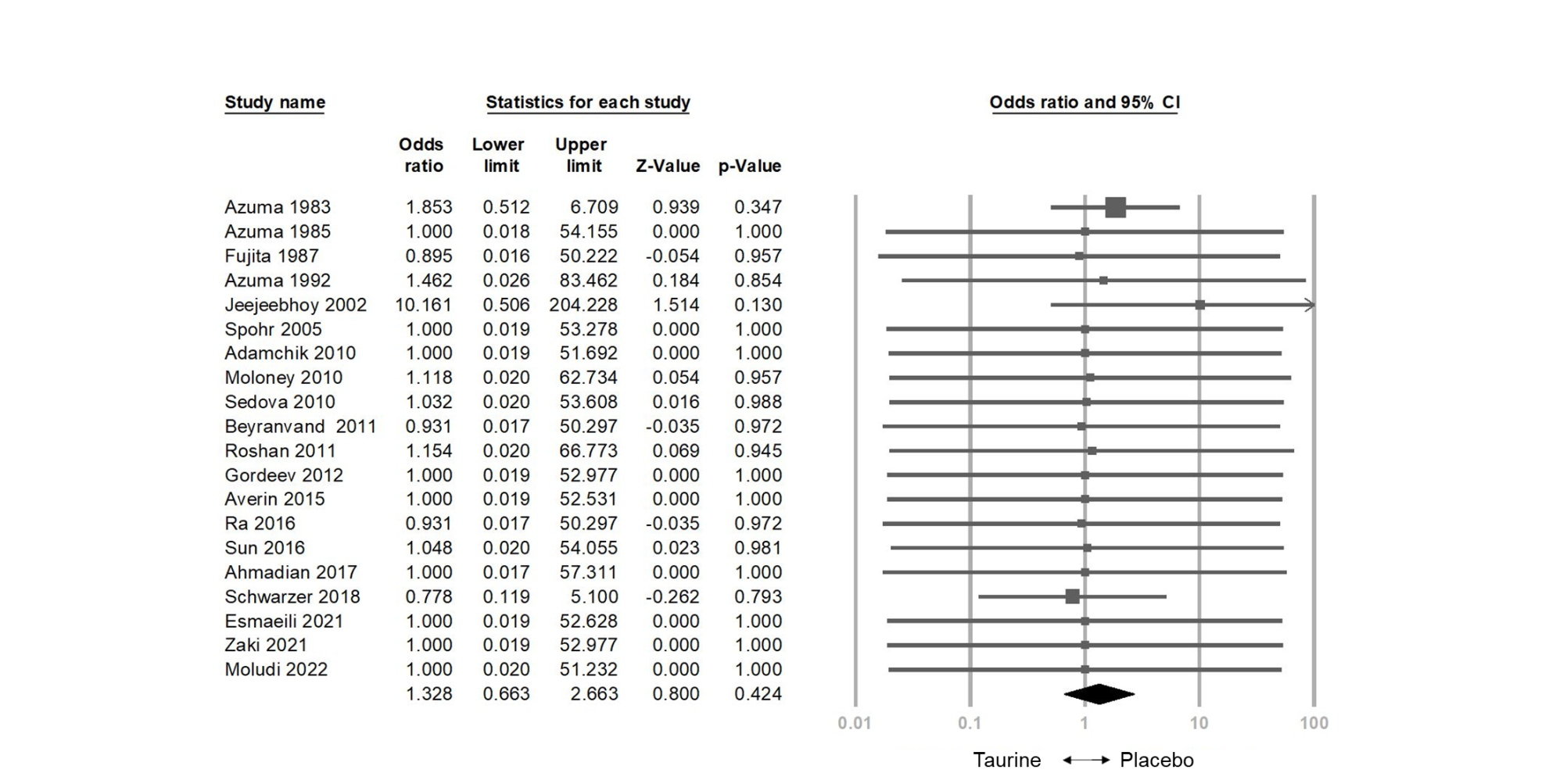
**

Heterogeneity: df=19 (*p*=1.000), *I*^2^=0.000
